# New early phenotypic markers for cucumber green mottle mosaic virus disease in cucumbers exposed to fluctuating extreme temperatures

**DOI:** 10.1038/s41598-021-98595-4

**Published:** 2021-09-24

**Authors:** Ori Molad, Elisheva Smith, Neta Luria, Noa Sela, Oded Lachman, Elena Bakelman, Diana Leibman, Aviv Dombrovsky

**Affiliations:** 1 Department of Plant Pathology and Weed Research, Agricultural Research Organization, The Volcani Center, 68 HaMaccabim Road, P.O.B 15159, 7505101 Rishon LeZion, Israel; 2 The Robert H. Smith Faculty of Agriculture, Food and Environment, The Hebrew University of Jerusalem, 761001 Rehovot, Israel

**Keywords:** Biotic, Environmental impact, Virus-host interactions

## Abstract

Studies of early stages of cucumber green mottle mosaic virus (CGMMV) disease have been recently focused on plant molecular responses. However, extreme diurnal environmental temperatures, characteristic of global climate changes, could affect plant susceptibility and disease phenotype progression. Our studies of CGMMV disease progression, under simulated extreme temperature waves, have revealed two new disease initiation phenotypes that developed gradually, preceding severe symptom manifestations of post-recovery CGMMV systemic infections. 'Early post-recovery stage' bright yellow islands (BYIs) with defined boundaries amid asymptomatic leaf blades were first emerging followed by 'late post-recovery stage' BYIs with diffused boundaries. A deduced CGMMV disease progression scheme, postulating BYI symptom occurrence time-windows, revealed BYIs in field grown cucumber plants exposed to extreme diurnal temperatures. Profiling ontology of cucumber differentially expressed genes in BYIs *vs* the associated dark-green surrounding tissues disclosed activation of jasmonic acid (JA) pathway in 'early post-recovery stage' BYIs. JA signaling was inactivated in 'late post-recovery stage' BYIs concomitant with increasing expressions of JA signaling inhibitors and downregulation of JA responsive phenylpropanoid pathway. Our results disclosed a new phenotypic description of CGMMV disease initiation, characteristic of cucumbers grown under extreme environmental temperature fluctuations. The BYI phenotypes could define a time-window for CGMMV disease management applications.

## Introduction

Cucumber green mottle mosaic virus (CGMMV) is a plant pathogenic virus belonging to the genus *Tobamovirus* in the *Virgaviridae* family. CGMMV causes severe disease symptoms in plants belonging to the family *Cucurbitaceae*, affecting important cucurbits such as cucumber (*Cucumis sativus*), muskmelon (*Cucumis melo*) and watermelon (*Citrullus lanatus*). CGMMV was first described in England in 1935 infecting cucumber plants, and since then it spread across Eurasia in an increasing rate, and reached North America, Africa and Australia in the second decade of the twenty-first century^1,2^. The worldwide spread of CGMMV is primarily the result of highly stable viral particles, which withstand extreme environmental conditions and are preserved in soil, plant debris and on plant growth facility's infrastructures^3^. CGMMV contaminated soil or seeds constitute a primary infection source that is followed by a massive secondary spread occurring mechanically during agro-technical practices (pruning, trellising), involving direct contact while handling the plants. The most prominent symptoms associated with CGMMV disease are foliar green mottling and mosaic patterns^2^.

Management of CGMMV disease distribution via hygienic conduct and agro-technical tool disinfections as well as plant debris virus inactivation, has been recently improved concomitant with the development of new disinfectants and applications of new combinations of strategies including grafting on various tolerant rootstocks^4,5^. However, attempts to prevent CGMMV primary infections by applying seed disinfection strategies are hampered by virus contamination of the perisperm-endosperm envelope, localized underneath the seed-coat specifically in cucurbits^6^. Therefore, a third important strategy for CGMMV disease control should involve treatments of infected plants at early disease stages with phyto-hormones, various biologically active molecules and/or double stranded RNA, the latter showed successful results against other tobamoviruses^7^. That third strategy attempts to promote plant resistance and alleviate symptom manifestations. A prerequisite for the third approach is mastering early manifestations of disease progression that are profoundly affected by environmental conditions, primarily the temperature. Host susceptibility, initiation of plant resistance mechanisms, viral RNA accumulation and RNA silencing machinery in various virus-infected plants are modified by temperature changes^8^. Interestingly, the various high temperature induced molecular mechanisms are sometimes associated with disease symptom recovery; early manifestations of dark green islands (DGIs) that are indicative of inhibition of viral accumulation in areas surrounded by systemic infections; or the opposite effect of overcoming resistance gene induced hypersensitive response followed by initiation of systemic infections^8–10^. Fluctuating environmental temperatures associated with global warming constitute new challenges for disease progression studies. Importantly, recent studies of diurnal temperature range (DTR) have shown a reversal from a decrease, which was associated with elevation of both high and low temperatures, to a statistically significant increase since the 1990s^11^. The combinations of increasing high temperatures and high DTRs expose field crops to fluctuating extreme temperatures that could affect viral disease manifestations. We have previously shown that in CGMMV infected cucumber plants grown at high temperatures severe disease manifestations of green mottling and mosaic were enhanced^12^. However, the contribution of fluctuating extreme temperatures to the high environmental temperature effect on viral disease symptom manifestations have not been studied yet. In our study, we have analyzed the effect of combined environmental conditions of high temperatures and fluctuating extreme temperature changes on disease progression in CGMMV infected cucumbers. The high temperature induced plant recovery allowed documentation of early phenotypes of CGMMV disease initiation at the post-recovery stage. We have found unique early features of CGMMV infection in cucumber plants under both glasshouse controlled conditions and field conditions. The new phenotypes were associated with distinct changes in differentially expressed plant genes and molecular pathways, which could be indicative of the preferable treatments for ameliorating plant resistance and alleviating symptom development. Importantly, our findings could define a stage during CGMMV disease symptom progression, showing unique phenotypic characteristics that mark the appropriate time-window for implementation of disease mitigation strategies in a commercial scale.

## Results

### Early CGMMV disease phenotypes identified in cucumbers following symptom recovery induced by an abrupt temperature raise

**Figure 1:**
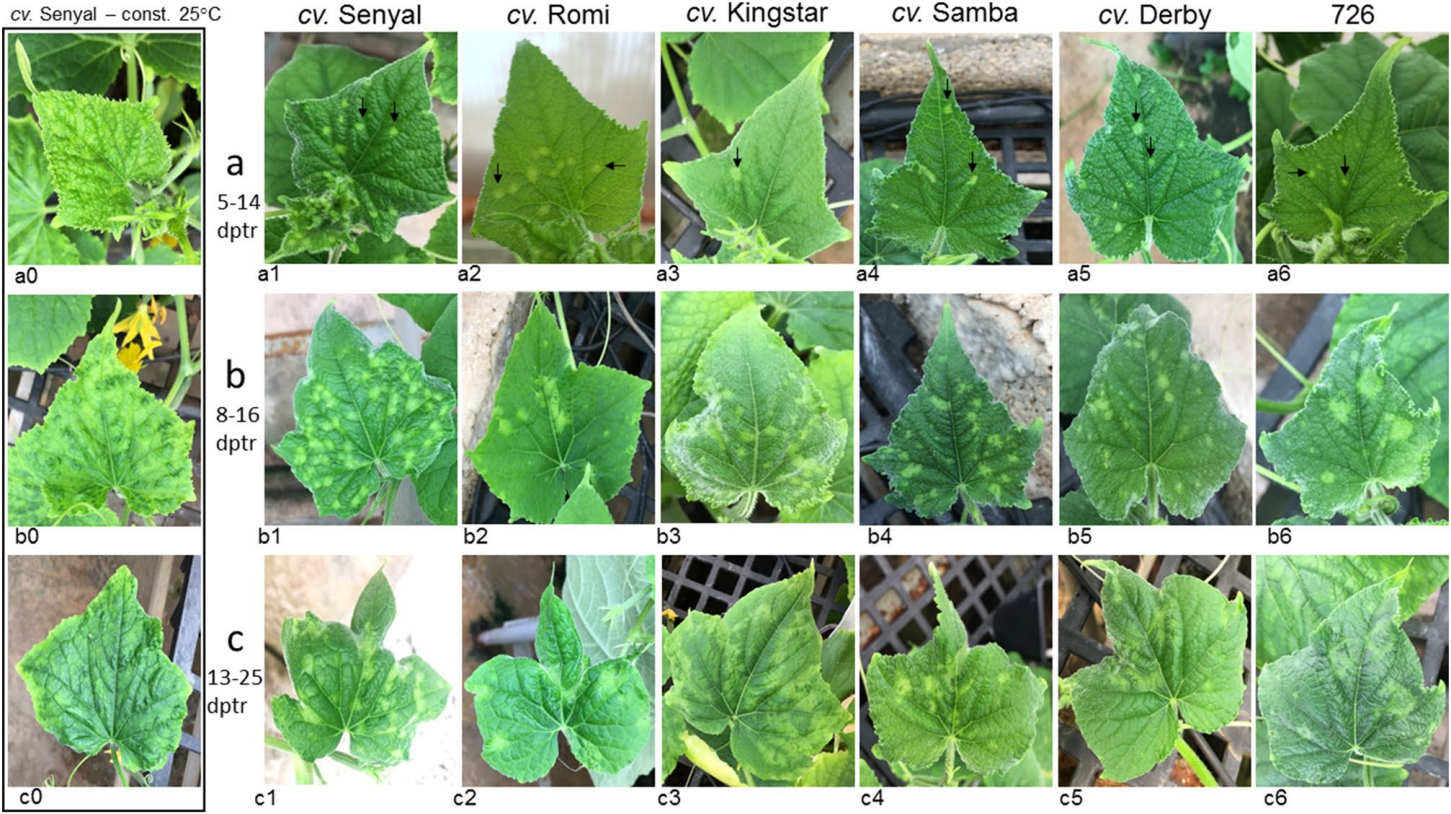
Development of 'bright yellow islands' (BYIs) and reemerging CGMMV disease symptoms on recovered cucumber leaves. (**a**) 'Early post-recovery stage' observed at 5–14 dptr. (**b**) **'**Late post-recovery stage' observed at 8–16 dptr. (**c**) 'Second symptomatic stage' observed at 13–25 dptr. (**a0–c0**) CGMMV-infected cucumber plants *cv*. Senyal kept at a constant 25 °C growth chamber showing mottling mosaic symptoms. (**a1–a6**) BYIs at the 'early post-recovery stage' showing defined boundaries. (**b1–b6**) BYIs at the 'late post-recovery stage' showing diffused boundaries. (**c1–c6**) Reemerging symptoms of the combined phenotype of yellow patched mottling and mosaic on slightly deformed leaves at the 'second symptomatic stage'. (**a1**, **b1**, **c1**) *cv*. Senyal; (**a2**, **b2**, **c2**) *cv*. Romi; (**a3**, **b3**, **c3**) *cv*. Kingstar; (**a4**, **b4**, **c4**) *cv*. Samba; (**a5**, **b5**, **c5**) *cv*. Derby; (**a6**, **b6**, **c6**) 726. dptr, days post temperature raise; black arrows marked 'early post-recovery stage' BYIs.

High growing temperatures have been associated with the occurrence of disease symptom recovery of virus infected plants (Supplementary Fig. S1). We have now studied the effect of an abrupt temperature raise on phenotypic characteristics of CGMMV disease that reemerged following plant recovery. Nine different cucumber cultivars, inoculated with CGMMV, were grown at 25 °C ± 2 and at seven days post inoculation (dpi), prior to symptom development, the temperature was rapidly raised to 32 °C ± 2 (reaching the set temperature in 15 min). Symptom manifestations of mottling interlaced with yellow dots or patches were observed on young leaves next to the apical leaves 5–8 h post temperature raise (hptr) (Supplementary Fig. S1). A close follow-up of disease progression carried out on six cultivars showed that at 48 hptr all the tested plants in each of the cultivars were systemically infected, showing ELISA optical density (OD) value range of 0.62–2.6 (with 0.08 negative reference [NR] OD values) (Supplementary Table S1). The progression of the systemic infection (3–5 leaves below the apical leaf per plant) was observed in older leaves concomitant with the new developing apical leaves. CGMMV disease symptom recovery was observed in each of six tested cultivars but time lengths between the systemic phenotype manifestations, induced by the temperature raise, and emergence of asymptomatic leaves differed between the tested cucumber cultivars. The earliest recovery was observed in *cv*. Romi at 11 dpi/4 days post temperature raise (dptr) and the latest recovery occurred in *cv*. Kingstar (KS) at 15 dpi/8 dptr (Supplementary Table S1). Two to three days post CGMMV disease symptom recovery, the asymptomatic leaves developed a new and unique phenotype named 'bright yellow islands' (BYIs), with defined boundaries amid asymptomatic surrounding dark green tissues (Fig. 1a1–a6). The disease stage of the early BYI manifestations during the gradual CGMMV disease reemergence was defined as the 'early post-recovery stage'. The leaf tissue surrounding BYIs remained visually asymptomatic for at least two days (in *cv.* SN). Following that time new BYIs showing diffused boundaries were developed (Fig. 1b1–b6). This stage was defined as a ‘late post-recovery stage’. The early and late post-recovery stage BYIs were detected on the upper leaves, below the apical leaf, and seldom were detected on the apical leaf that remained asymptomatic. Importantly, BYI phenotypic stages were not observed in systemically infected CGMMV inoculated cucumber plants that were kept at 25 °C ± 2 (Fig. 1a0–c0) and no distinct BYI manifestations were apparent in CGMMV-infected plants grown at a constant temperature of 32 °C ± 2 during a post-recovery disease progression (Supplementary Fig. S2). By 21 dpi/14 dptr (in *cv*. SN) the newly described symptomatic leaves showed a combined phenotype of yellow patched mottling and mosaic, occasionally accompanied by apical leaf deformations, defining a ‘second symptomatic stage’ of CGMMV systemic infection (with ELISA OD value range of 0.35–1.24 [0.01 NR OD values]) (Fig. 1c1–c6). Importantly, the early and late post-recovery stage BYIs were detected on 2–3 leaves per plant (at the 7–8 leaf stage), in all the plants of five of the tested cultivars (a total of 163 plants). The cucumber plants of *cv*. Romi showed BYI manifestation in up to 5 leaves per plant, in all the inoculated plants (10 plants), which could indicate a high level of synchronization of disease initiation or a lower rate of disease progression and increasing BYI persistence (Supplementary Table S1).

### 'Bright yellow islands' (BYIs) indicate the initiation of CGMMV disease reemergence

**Figure 2:**
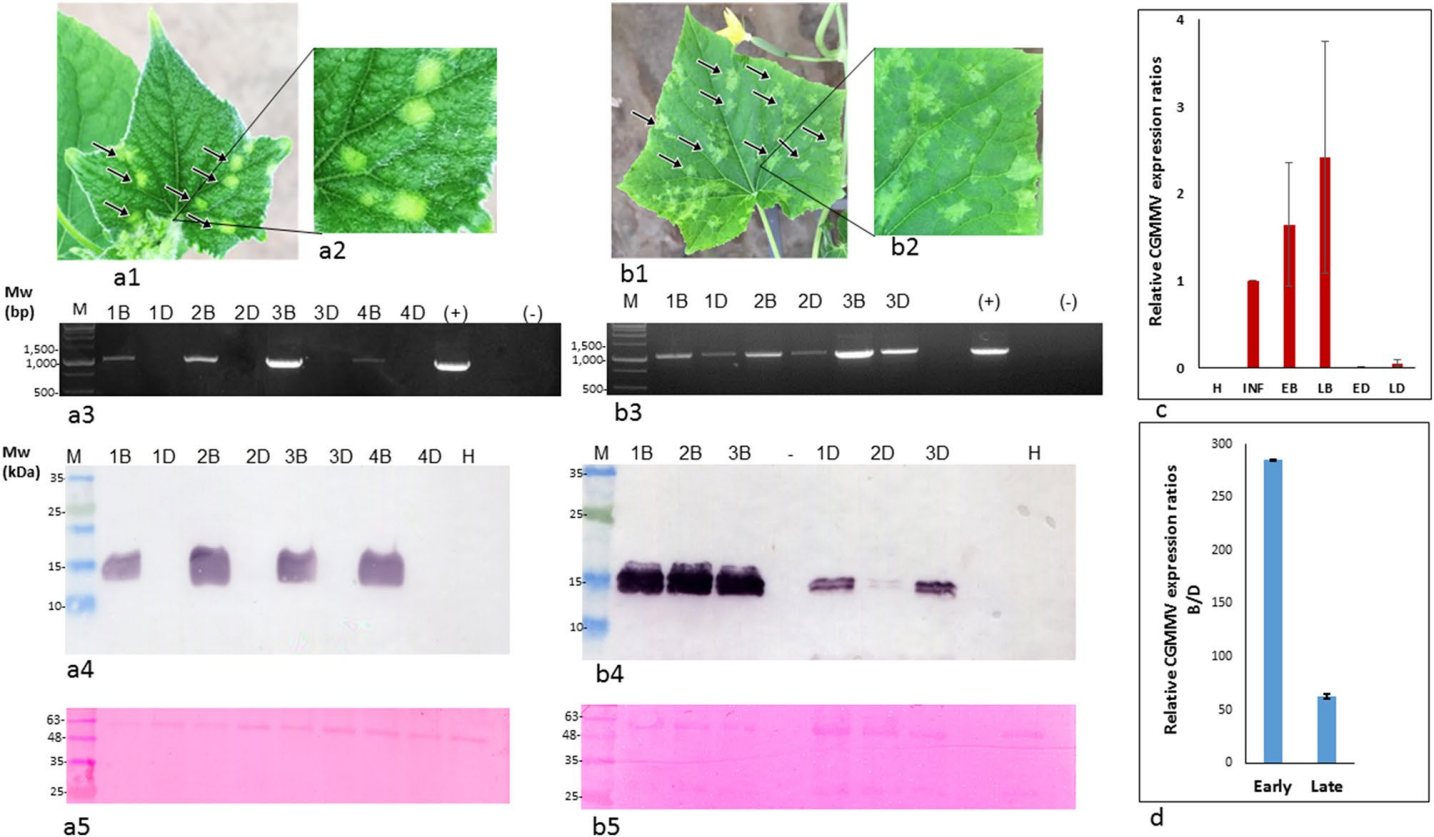
CGMMV in ‘bright yellow islands’ (BYIs) and the corresponding dark green surrounding tissues of cucumber leaves at early and late post-recovery stages. (**a1**) A cucumber leaf (*cv*. Senyal) showing 'early post-recovery stage' BYIs. (**a2**) A close-up on the 'early post-recovery stage' BYIs. (**a3**) RT-PCR detecting CGMMV in sampled 'early post-recovery stage' BYIs but not in the corresponding dark green surrounding tissues. (**a4**) Western blot analyses showing CGMMV coat protein in sampled 'early post-recovery stage' BYIs but not in the corresponding dark green surrounding tissues. (**a5**) A Ponceau S stained membrane showing total loaded proteins in all lanes. (**b1**) A cucumber leaf (*cv*. Senyal) showing 'late post-recovery stage' BYIs. (**b2**) A close-up on the 'late post-recovery stage' BYIs. (**b3**) RT-PCR detecting CGMMV in sampled 'late post-recovery stage' BYIs and the corresponding dark green surrounding tissues. (**b4**) Western blot analyses showing CGMMV coat protein in sampled 'late post-recovery stage' BYIs and the corresponding dark green surrounding tissues. (**b5**) A Ponceau S stained membrane showing total loaded proteins in all lanes. B, bright yellow islands; D, the associated dark green surrounding tissues; M, molecular size marker; H, healthy control; (+), positive control; (−), negative control. Black arrows mark the BYIs. (**c**) RT-qPCR analyses of relative CGMMV expression in BYIs and the corresponding dark green surrounding tissues of early and late post-recovery stages (± SD). H, healthy control; INF, a CGMMV infected symptomatic cucumber leaf; EB, 'early post-recovery stage' BYIs; LB, 'late post-recovery stage BYIs; ED, 'early post-recovery stage' dark green surrounding tissues; LD, 'late post-recovery stage' dark green surrounding tissues. (**d**) Relative CGMMV expression ratios between BYIs and the associated dark green surrounding tissues (Bright/Dark, ± SD) at the early and late post-recovery CGMMV disease stages.

CGMMV infected cucumber leaves at both early and late post-recovery stages were tested for differential viral expression between the BYIs and the corresponding dark green surrounding tissues (Fig. 2). At the 'early post-recovery stage', RT-PCR analyses showed undetectable virus titer in the dark green asymptomatic tissues, compared to the associated BYIs (a total of 5 samples were tested) (Fig. 2a2). Similar results were obtained by western blot analyses, showing CGMMV coat protein (CP) in the BYIs, whereas the corresponding asymptomatic dark green surrounding tissues were devoid of the virus (a total of sixteen samples were tested) (Fig. 2a3–a4). In contrast, RT-PCR and western blot analyses of CGMMV at the 'late post-recovery stage' showed that CGMMV was present in both the BYIs, with the diffused boundaries, and the corresponding dark green surrounding tissues (Fig. 2b2–b4). These results could indicate that CGMMV in 'early post-recovery stage' BYIs with defined boundaries overcame plant resistance when 'late post-recovery stage' BYIs were manifested. The increase in CGMMV titer in the dark green surrounding tissue associated with 'late post-recovery stage' BYIs compared to the asymptomatic dark green tissue surrounding 'early post-recovery stage' BYIs was confirmed by RT-qPCR (Fig. 2c). Importantly, whereas viral content in early and late post-recovery stage BYIs were generally comparable, the calculated relative CGMMV expression ratios between the BYIs and the associated dark green surrounding tissues (B/D) showed a prominent decrease upon progression from the early to the late post recovery stages (Fig. 2d).

### A time-table scheme of 'Bright yellow island' manifestations, challenged in field cultivated cucumber plants

**Figure 3:**
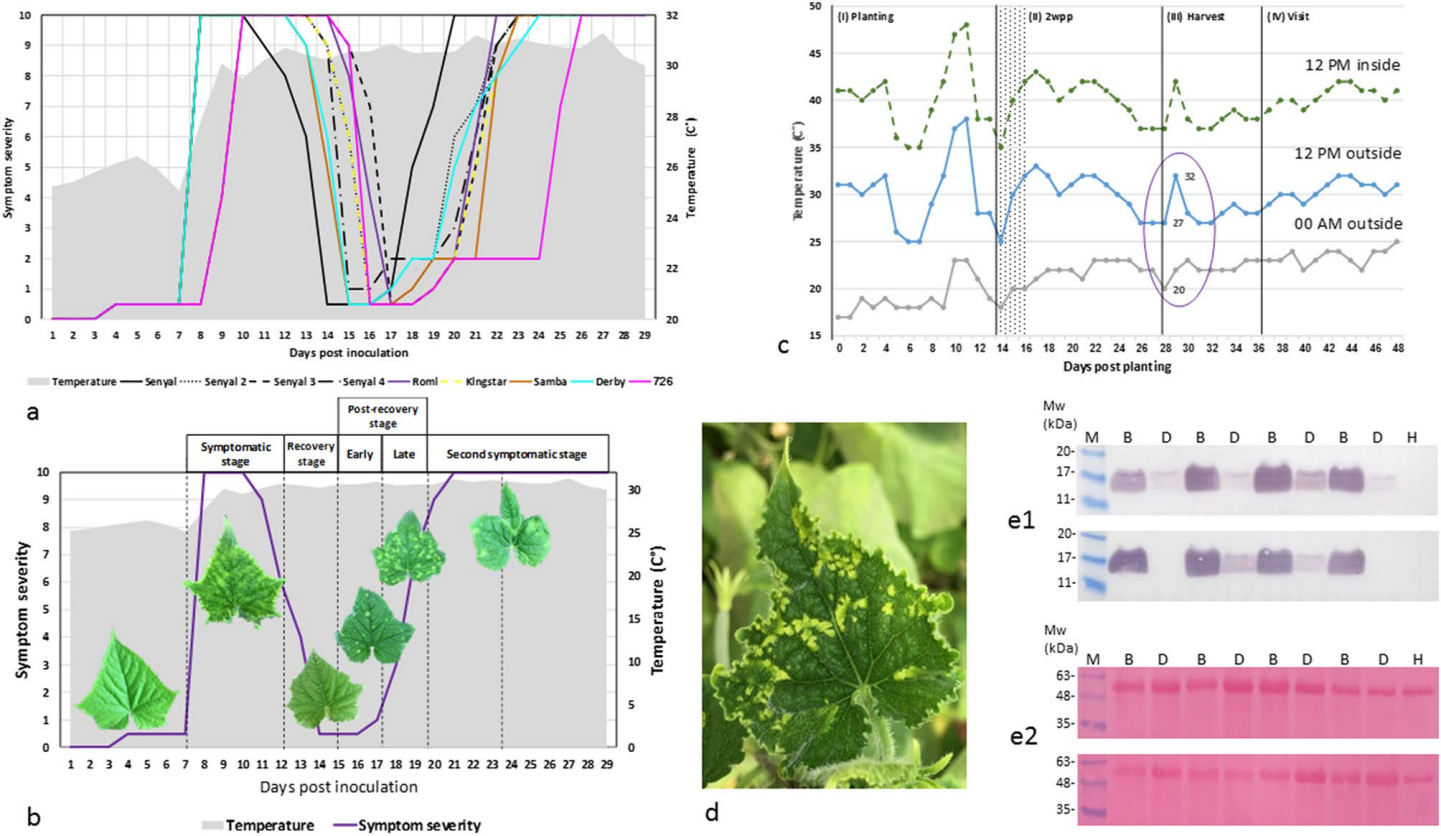
Establishing a CGMMV disease-phenotype progression scheme for ‘bright yellow island’ (BYI) development and its manifestation under natural environmental temperature conditions. (**a**) A graphical summary of CGMMV disease symptom manifestations during the gradual disease progression in the various tested CGMMV inoculated cucumber plants. Six different cultivars (7–60 plants in the various experiments) were subjected to an abrupt temperature raise from 25 ± 2 °C to 32 ± 2 °C at the pre-symptomatic stage, 7 days post inoculation. Plants were inspected daily. Temperature logging was automatic, at hourly intervals, marked by a grey area. The various *cv*. line symbols are: Senyal (4 replications), black symbols of a solid line, a dashed line, a long dashed-dot line and a round dot line ; Romi; a purple solid line; Kingstar; a yellow square dotted line; Samba, an orange solid line; Derby, a light blue solid line and 726, a pink solid line. (**b**) An integrated CGMMV disease progression scheme illustrating periodical symptom manifestations following the abrupt temperature raise, marked by a purple line. A grey line marks the monitored temperatures. Symptom severity ratings: 0–1: no visible symptoms; 2: bright yellow islands; 3–7: slight to medium severity showing a combined phenotype of yellow patched mottling; 7 and higher: severe symptoms of yellow patched mottling and mosaic on slightly deformed leaves associated with systemic CGMMV infection (ELISA OD value range of 0.26–1.22, negative reference OD: 0.01). (**c**) The BYI manifestation time-table postulated BYI detections in cucumber plants cultivated in a commercial net-house following a heat wave (marked by a purple ellipse). Recorded high and low environmental temperatures were marked by blue and grey lines, respectively. Recorded temperatures inside the greenhouse were marked by a dotted (green) line. I, a presumed primary infection at planting; II–III, a presumed secondary spread upon trellising and harvest; wpp, weeks post planting. (**d**) A cucumber leaf from the commercial greenhouse showing BYIs. (**e1**) Western blot analyses of 8 selected plants, collected from 4 different commercial net-houses, showing various stages of BYI development. (**e2**) detection of loaded proteins by Ponceau S staining. B, bright yellow islands; D, corresponding dark green surrounding leaf tissues; H, negative control; M, molecular weight marker.

The gradual CGMMV disease symptom manifestations following cucumber plant recovery, observed in all the tested cucumber cultivars that were subjected to an abrupt temperature raise, served for drawing a time-table scheme for disease progression (Supplementary Table S1, Fig. 3a,b). The gathered data specified time-windows for five phenotypic stages of CGMMV disease in cucumbers following an extreme temperature raise (25 ± 2 °C to 32 ± 2 °C). Time lengths between the abrupt temperature raise and severe symptom induction ranged between 5 to 48 hptr. Time lengths before reaching symptom recovery ranged between an additional 4 days (d) to 8 d. Early and late post-recovery stage BYIs appeared 2–3 d following the recovery stage. The consequential 'second symptomatic stage', showing the combined phenotype of yellow patched mottling and mosaic, appeared after additional 4–7 d. Applying the derived CGMMV disease phenotype progression scheme to field cultivated cucumber plants revealed occurrence of BYIs following natural heat wave events. The majority of the commercial cucumber net-houses in Ahituv, Israel (32° 23′ 19.4″ N 34° 59′ 22.4″ E) are frequently infected with CGMMV during the three continuous yearly growing cycles. A visit to a selected commercial cucumber net-house in Ahituv, 8 days following a heat wave, disclosed the BYI phenotype in five plants, found in a cluster of *ca*. fifty symptomatic plants that were amid asymptomatic surroundings (Fig. 3c,d). In addition, unscheduled visits to 3 Ahituv commercial net-houses during early summer weeks disclosed the manifestations of BYIs at various stages (Fig. 3e). Amid 10,000 plants in each of 2 of the net-houses, at 30 days post planting (dpp), 20–30% of the plants that were symptomatic in old leaves showed recovery in apical leaves. In those net-houses BYIs were detected in 0.1–< 1% of the plants. In a third 10,000 plant net-house, which showed 50–60% recovered plants at 45 dpp, 5–10% of the plants showed BYI manifestations. BYIs and the corresponding dark green surrounding tissue samples, dissected from 16 young leaves collected from 16 different infected plants, were subjected to western blot analyses. Several stages of BYI manifestations were apparent, showing various titers of CGMMV-CP in the surrounding dark green tissues associated with the BYIs (Fig. 3e1, 2).

### Post-recovery stage BYIs are early indicators of CGMMV systemic infection potential

In order to ascertain the possible role of the BYIs in CGMMV systemic infection we have tested the infectious potential of early and late post-recovery stage BYIs in biological assays by inoculating cucumber plants *cv*s. SN and Noname. CGMMV inoculum sources applied onto *cv*. SN plants were extracted from either the 'early post-recovery stage' BYIs (five islands) or the entire associated dark green surrounding leaf tissue of a CGMMV-infected cucumber leaf (to ensure an infectious potential test for the lowest CGMMV content). CGMMV inoculum sources applied onto *cv*. Noname plants were extracted from equally weighed samples of 'late post-recovery stage' BYIs and the associated dark green surrounding tissues. The inoculated plants were grown in temperature controlled chambers of 25 °C and 32 °C. Importantly, 'early post-recovery stage' BYI inoculum source caused CGMMV systemic infection in 10/10 inoculated plants (Table 1). The low virus titer in the 'early post-recovery stage' entire dark green asymptomatic tissue of the CGMMV-infected leaf clearly comprised a CGMMV infectious source, causing disease symptom manifestations and systemic infection in 7/10 inoculated SN plants. In addition, a recovery stage characteristic of 32 °C growing temperatures was apparent in the inoculated plants at 13 dpi (Table 1). Progression of CGMMV systemic infection towards increasing CGMMV titer in the dark green surrounding tissue associated with 'late post-recovery stage' BYIs was clearly reflected in the infectious ratios of the equally weighed inoculum source samples applied onto *cv*. Noname plants. At 25 °C growing conditions, at 14 dpi, the BYI and the associated dark green surrounding tissue inoculum sources caused systemic infection in 15/15 and 7/15 inoculated Noname plants, respectively (Table 1). In addition, a recovery stage was observed in *cv*. Noname inoculated plants at 32 °C growing temperatures, at 13 dpi as well.

**Table 1:** An infectious potential of CGMMV in BYIs and the corresponding dark green surrounding tissues of early and late post-recovery stages, determined in biological assays.

CGMMV inoculum source	Temp (°C)	Symptom emergence (dpi)	Recovery (dpi)	Symptom reemergence (dpi)	Sampling day (dpi)	Infected plants (ratio)	ELISA positive OD range
'Early' BYIs (5 discs, 3 mm/disc, 28 mg)	25	10	None	na	27	5/5	0.3–0.4
	32	10	13	19	22	5/5	0.3–0.5
'Early' total associated DST (~ 75 cm^2^,1.4 gr)	25	10	None	na	27	3/5	0.3–0.5
	32	10	13	19	22	4/5	0.3–0.8
'Late' BYIs (4 discs, 20.8 mg)	25	9	None	na	14	15/15	0.23–0.25
	32	9	13	18	10	12/15	0.21–0.31
'Late' associated DST (4 discs, 20.2 mg)	25	9	None	na	14	7/15	0.23–0.25
	32	9	13	18	10	8/15	0.20–0.31

*'Early'* early post-recovery stage, *'Late'* late post-recovery stage, *DST* dark surrounding tissues, *dpi* days post inoculation, *na* not applicable, ELISA negative reference OD range was 0.06–0.09; in brackets, numbers of collected discs, total disk weights and sizes of discs or leaf area.

### Differential cucumber gene expressions in BYIs of early and late post-recovery stages compared to the corresponding dark green surrounding tissues

**Figure 4:**
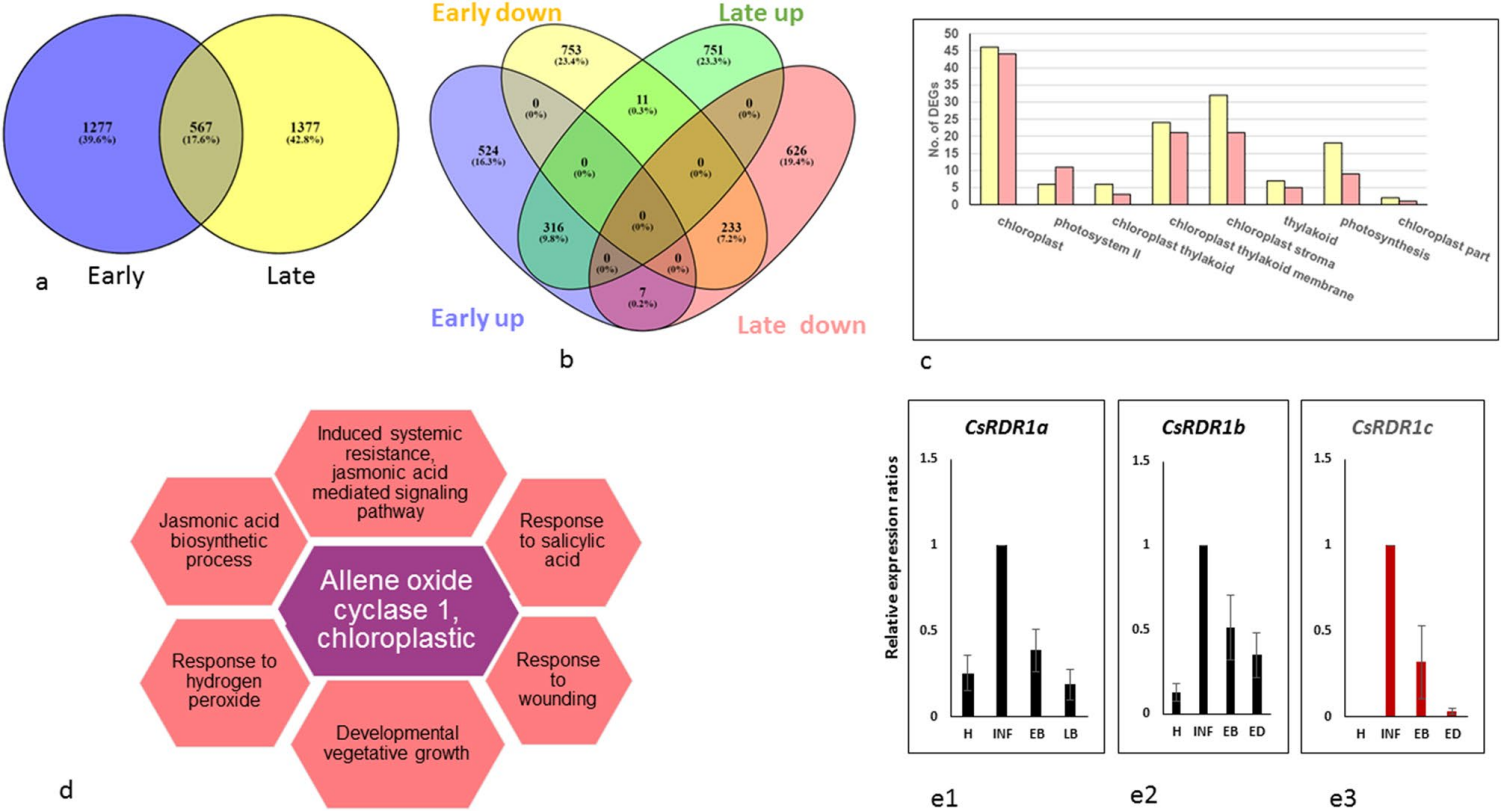
Differentially expressed genes (DEGs) and selected annotated cellular processes in bright yellow islands (BYIs) of CGMMV infected cucumber leaves at early and late post-recovery stages compared to the corresponding dark green surrounding tissues. (**a**) Venn diagram showing the overlap between DEGs in BYIs of CGMMV infected cucumber leaves at the early and late post-recovery stages compared to the corresponding dark green surrounding tissues. (**b**) Venn diagram showing both unique and overlapping distribution of up-regulated and down-regulated DEGs found in the BYIs at the early and late post-recovery stages compared to the corresponding dark green surrounding tissues. Early, 'early post-recovery stage' BYIs; Late, 'late post-recovery stage' BYIs; up, upregulated DEGs; down, downregulated DEGs. (**c**) Distribution of 219 DEGs out of the total 233 downregulated DEGs, shared by early and late post-recovery stage BYIs, among the various annotated cellular processes associated with the photosynthesis pathway. Yellow colored columns, no. of DEGs downregulated in the 'early post-recovery stage' BYIs; pink colored columns, no. of DEGs downregulated in the 'late post-recovery stage' BYIs. (**d**) Depiction of selected cellular pathways annotated for allene oxide cyclase one, one of seven DEGs upregulated in ‘early post-recovery stage’ BYIs and downregulated in ‘late post-recovery stage’ BYIs. (**e1–3**) RT-qPCR analyses showing relative gene expression ratios of *CsRDR1a*, *CsRDR1b* and *CsRDR1c* genes in early and late post-recovery stage BYIs and the associated dark green surrounding tissues. H, healthy control; INF, a CGMMV systemically infected cucumber leaf; EB, 'early post-recovery stage' BYIs; LB, 'late post-recovery stage' BYIs; ED, 'early post-recovery stage' dark green surrounding tissues; LD, 'late post-recovery stage' dark green surrounding tissues.

CGMMV disease progression from the initial infectious titer, mostly restricted to the 'early post-recovery stage' BYIs, to the increasing infection of the dark green tissue surrounding the 'late post-recovery stage' BYIs, could be associated with modified plant molecular pathways critical for a successful defense response. Differential gene expression between BYIs and their associated dark green surrounding tissues was analyzed in extracted total RNA containing the full length CGMMV genome, sequenced by HTS, with 99.8% genome coverage and 136–156 read depths (Supplementary Fig. S3, Supplementary Table S2). Analyses of the differential cucumber gene expression revealed 1844 and 1944 differentially expressed genes (DEGs) in the BYIs of early and late post-recovery stages, respectively, with 567 common genes (Fig. 4a). In ‘early post-recovery stage’ BYIs, 997 DEGs were significantly down-regulated and 847 DEGs were significantly upregulated (α < 0.05). In ‘late post-recovery stage’ BYIs, 866 DEGs were significantly down-regulated and 1078 DEGs were significantly upregulated (α < 0.05) (Fig. 4b). The BYIs at the two stages shared 233 downregulated DEGs and 316 upregulated DEGs. Gene ontology (GO) enrichment analyses of the identified DEGs showed that as expected, among the 233 downregulated DEGs, shared by BYIs of early and late post-recovery stages, significantly downregulated 219 DEGs were involved in 8 enriched GO categories related to photosynthesis pathway or chloroplasts (Supplementary Table S3, Fig. 4c). Importantly, seven common DEGs that were upregulated in ‘early post-recovery stage’ BYIs were downregulated in ‘late post-recovery stage’ BYIs and eleven DEGs had the opposite characteristics (Fig. 4b, Supplementary Table S4). GO analysis results of the seven common DEGs, upregulated in 'early post-recovery stage' BYIs and downregulated in 'late post-recovery stage' BYIs, revealed the involvement of allene oxide cyclase I that participates in jasmonic acid (JA) synthesis pathway. Several of the GO processes associated with allene oxide cyclase I and the JA signaling pathway are listed in Fig. 4d.

Upregulation of the JA signaling in 'early post-recovery stage' BYIs was associated with a concurrent activation of genes responsive to JA. A range of molecular pathways associated with JA signaling involves JA-induced R2R3 MYB transcription factors^13^. In 'early post recovery stage' BYIs one gene of bHLH-MYC and R2R3-MYB transcription factor was differentially upregulated and 'late post-recovery stage’ BYIs showed downregulation of one gene and upregulation of two genes. JA-induced R2R3-MYB could mediate the following effects: (1) activation of RNA silencing; (2) enhancement of basal reactive oxygen species (ROS) via activation of l-ascorbate oxidase (AO); (3) activation of phenylalanine ammonia-lyase (PAL), which is a key regulatory enzyme in the phenylpropanoid pathway that is associated with plant resistance against viral infections^14–16^. Regarding RNA silencing pathway we have found that specifically at the 'early post-recovery stage', the BYIs showed differential upregulation of RNA dependent RNA polymerase (RDR) (Supplementary Table S3). We have confirmed by RT-qPCR that unlike RDR1a and RDR1b, which were expressed in both healthy and infected plants, the RDR1c, which was specifically associated with cucumber plant RNA silencing response to viral infections^17^, was significantly upregulated in 'early post-recovery stage' BYIs compared to the associated dark green surrounding tissue (Fig. 4e). Concomitantly, endoribonuclease Dicer homolog 1 and protein ARGONAUTE 1 (AGO1), involved in post transcriptional gene silencing, were differentially upregulated in both early and late post-recovery stage BYIs (Supplementary Table S3).

Regarding ROS activation, expressions of genes of the ROS scavenger thioredoxin, which mediates virus derived small interfering RNA (vsiRNA) effects on ROS accumulation^18^, were differentially downregulated at both early and late post-recovery stages (Supplementary Table S5). In addition, two AO genes were differentially upregulated in the 'early post-recovery stage' BYIs and four AO genes were differentially upregulated in the 'late post-recovery stage' BYIs (supplementary Table S5). Differential expression profiles of additional enzymes involved in plant response to oxidative stress upon various viral infections, are summarized in Supplementary Table S5^19^. Unlike the above positive response of the cucumber plant genes to JA-induced R2R3-MYB, PAL expression was downregulated in 'early post-recovery stage' BYIs (one gene), as compared to the associated dark green surrounding tissue. In addition, several JA signaling inhibitory molecules were upregulated already at 'early post-recovery stage' BYIs. A group of proteins associated with plant growth regulation, the SQUAMOSA promoter-binding-like (SPL) proteins, as well as several WRKY transcription factors, found to be modulated upon plant engagement of defense response^20^, were among the 316 upregulated DEGs shared by early and late post-recovery stage BYIs (Supplementary Tables S6, S7). Importantly, SPL9 that negatively regulates JA signaling^21^, was upregulated in 'early post-recovery stage' BYIs. Similarly, WRKY transcription factors that participate in JA signaling inhibition^22^, were differentially upregulated in BYIs of early and late post-recovery stages. WRKY 51 was upregulated at the 'early post-recovery stage', whereas WRKY 46 and WRKY 53 were specifically upregulated in the 'late post-recovery stage' BYIs, where JA signaling was downregulated (Supplementary Tables S6, S7). Interestingly, in the 'late post-recovery stage' BYIs, expressions of six PAL genes were downregulated, compared to the associated dark green surrounding tissues. The central role of the phenylpropanoid pathway in plant resistance to viral infections led us to study the possibility that a prominent effect on the phenylpropanoid pathway activity was associated with CGMMV disease progression from early to late post-recovery stage BYIs and the initiation of a systemic infection. We have therefore analyzed the pathway enrichment of the DEGs at the two stages.

### Pathway enrichment analyses

In order to identify statistically significant cellular pathways associated with CGMMV disease initiation in cucumber plants showing the early and late post-recovery stage BYI phenotypes, the annotated biological processes of the DEGs at the two stages served for pathway enrichment analyses (Table 2, Supplementary Table S8). In the ‘early post-recovery stage’ BYIs carbon assimilation and TCA cycle were upregulated whereas –3,8 divinyl-chlorophyllide a biosynthesis, an intermediate metabolite in chlorophyll a synthesis pathway^23^, was downregulated. Importantly, upregulation of hydroxylated fatty acid biosynthesis, which could be associated with host lipid engagement in assembly of (+)RNA virus replication machinery^24^, was observed specifically in the ‘late post-recovery stage’ BYIs. Most-important were the findings that the phenylpropanoid pathway and scopolin and esculin biosynthesis, directly associated with scopoletin reported negative effect on tobamovirus replication as well as its reactive oxygen intermediate (ROI) scavenging activity^25^, were downregulated specifically in the ‘late post-recovery stage’ BYIs, compared to the associated dark green surrounding tissues. Further support for the possible crucial role of the phenylpropanoid pathway in cucumber plant response to CGMMV early disease stages came from comparisons of each of the early disease phenotypes to healthy cucumber plants (Supplementary Table S9). In the dark green tissue surrounding the 'late post-recovery stage' BYIs five PAL genes were upregulated, compared to healthy plants, and biosynthesis pathway of suberin monomers (involving the phenylpropanoid pathway) was upregulated. In addition, a concomitant upregulation of Calvin-Benson–Bassham cycle of carbon fixation occurred in the 'late post-recovery stage' dark green tissue whereas in the associated BYIs JA biosynthesis was downregulated, conferring to molecular pathways activated in the 'late post-recovery stage' dark green tissue an important role in plant response to initiation of a systemic CGMMV infection.

**Table 2:** Pathway enrichment analyses of differentially expressed genes in bright yellow islands of early and late post-recovery stages compared to the corresponding dark surrounding tissues.

Group	Cellular pathway	Expression	p value	FDR	No. of DEGs
'Early post-recovery stage' BYIs	Super pathway of cytosolic glycolysis (plants), pyruvate dehydrogenase and TCA cycle (PWY-5464)	Up-regulated	0.0000584	0.0066	12
	C4 photosynthetic carbon assimilation cycle, NAD-ME type (PWY-7115)		0.000496	0.028	8
	TCA cycle II (plants and fungi) (PWY-5690)		0.0011	0.0299	6
	Photosynthetic carbon assimilation cycle, PEPCK type (PWY-7117)		0.000886	0.0299	7
	3,8- divinyl-chlorophyllide a biosynthesis I (aerobic, light-dependent) (CHLOROPHYLL-SYN)	Down-regulated	0.0048	0.0000292	7
'Late post-recovery stage' BYIs	PWY-6433 hydroxylated fatty acid biosynthesis (plants)	Up-regulated	0.0297	0.00028	6
	PWY-7186 super pathway of scopolin and esculin biosynthesis	Down-regulated	0.0142	0.000183	6
	PWY1F-467 phenylpropanoid biosynthesis, initial reaction		0.0142	0.000116	6

*BYIs* bright yellow islands, *FDR* false-discovery rate, *DEGs* differentially expressed genes.

## Discussion

Tobamovirus-induced diseases of vegetable and ornamental crops are easily spread and affect the economy of countries and private growers worldwide. CGMMV has a highly significant ill-effect on cucurbit cultivation^2^. Importantly, there are only scarce reports on CGMMV resistant cucurbit cultivars, which are not yet available globally^26–28^. Although the various manifestations of CGMMV disease symptoms have been described since the virus discovery in 1935^1^, infected cucurbit plants exposed to fluctuating extreme temperatures, characteristic of the global climate change, could show significant new disease traits as well as different paces of symptom progression. In addition, recent studies of cucurbit transcriptome and microRNA profiles induced by CGMMV infections^29–32^ as well as profiles of vsiRNAs, regulating plant gene expressions^33,34^, have emphasized the necessity of a precise description of the early stages of CGMMV disease establishment.

A comprehensive study of annual DTRs in various countries over the past several decades has shown a trend toward an increasing temperature range, documenting statistically significant higher annual DTRs in Europe since the 1990s^11^. The impact of cucumber plant exposure to extreme temperatures, daily, on CGMMV disease symptom initiation and progression, was the subject of our current study. The inoculum source for our studies was a frozen lyophilized material of CGMMV-Rd, sequenced using SOLiD NGS platform that showed no other cucurbit infecting viruses in the preparation, and was also tested negative for various insect transmitted viruses by RT-PCR. The plants, subjected to an abrupt temperature raise from 25 to 32 °C, exhibited a systemic symptom development of mottling interlaced with bright yellow dots or patches, at 5–48 hptr (depending on the tested cultivar). That rapid response reaffirmed our previous observations of a direct correlation between increasing growing temperatures of CGMMV infected cucumber plants and a high rate of symptom manifestations^12^. In addition, the short time-span of temperature raise (15 min) seemed to assure synchronization of disease initiation stages that were not observed in field grown cultivars exhibiting various stages of BYI manifestations at the visit time (Fig. 3e1,2; see below). Following a recovery stage, induced by high temperatures, disease symptom reemergence in the inoculated cucumber plants revealed two distinct early symptoms of BYIs amid asymptomatic dark green surrounding tissues. Importantly, we have found that the observed different features of 'early post-recovery stage' BYIs, with defined boundaries, and 'late post-recovery stage' BYIs, with diffused boundaries, were indicative of disease progression towards systemic infection (Table 1). Accordingly, the dark green tissue surrounding 'early post-recovery stage' BYIs showed very low virus titer, which was much higher and readily detectable in the dark green tissue surrounding the 'late post-recovery stage' BYIs (Fig. 2). In addition, ratios of CGMMV relative gene expression between the BYIs and the associated dark green surrounding tissues (B/D) during progression from early to late post-recovery stages, measured by RT-qPCR, showed a prominent reduction (Fig. 2). This tight correlation between B/D reduction and disease progression occurred at the narrow time window of initiation of CGMMV systemic infections under the tightly controlled experimental conditions. Importantly, viral titer in the BYIs was not significantly increasing upon progression from early to late post-recovery stages. Concurrent tests of CGMMV inoculated cucumber plants kept at constant temperatures of either 25 °C or 32 °C did not reveal the distinct BYI symptoms during disease progression towards the symptomatic systemic stage. A large scale study of six different cucumber cultivars, monitoring BYI manifestations induced by an abrupt temperature raise under tightly controlled conditions, served for designing a timescale scheme that will allow postulation of BYI manifestations under field conditions (Fig. 3a,b). Apparently, BYIs were detected in up to 10% of the symptomatic plants in commercial net-houses that showed 50–60% recovery following an extreme temperature wave (Fig. 3c,d). These findings were most important considering the unfavorable field conditions of multiple risks of viral infections due to trellising, fruit picking and routine pesticide spraying as well as possible effects on plant defense response due to plant exposure to varying micro-climate conditions associated with shading, different solar radiation angles and irregular light intensities and durations.

Our analyses of the DEGs in the early and late post-recovery stage BYIs, as compared to their associated dark green surrounding tissues, revealed the importance of two major pathways in the early post-recovery cucumber plant response to CGMMV infection. One pathway was JA signaling that was upregulated in the 'early post-recovery stage' BYIs and downregulated in the 'late post-recovery stage' BYIs. JA signaling in 'late post-recovery stage' BYIs was also significantly reduced when compared to healthy cucumber plants (Supplementary Table S9). Importantly, high temperature dependent preference of JA signaling activation has been shown in *Nicotiana tabacum* defense response towards CMV infection^9^. The second pathway was the phenylpropanoid biosynthesis, which was prominently downregulated in the 'late post-recovery stage' BYIs, indicating high activity of the pathway in the associated dark green surrounding tissues at that stage. The early plant response to CGMMV by activation of JA signaling in the 'early post-recovery stage' BYIs, compared to the associated dark green surrounding tissue, was accompanied by upregulation of RDR1c relative expression, reported to be specifically associated with cucumber plant RNA silencing response to viral infections^17^. Interestingly, although RNA silencing of infecting viruses was attributed to plant recovery^8^, which was the set point stage of our CGMMV disease initiation study, RDR1c relative gene expression ratio was upregulated in 'early post-recovery stage' BYIs when compared to the associated asymptomatic dark green surrounding tissues (Fig. 3e). The relative expressions of RDR1a and RDR1b that were expressed in healthy plants as well, was not significantly different between the BYIs and the associated dark green surrounding tissue. Concomitant upregulation of endoribonuclease Dicer homolog 1 and AGO 1, as well as downregulation of thioredoxin, responsive to vsiRNAs^18^, could indicate JA activation of RNA silencing in the BYIs at that early disease stage^14^. In addition, the early thioredoxin downregulation and AO upregulation that could lead to ROS accumulation as well as the associated activation of plant immunity and antiviral response^35^ (Supplementary Tables S3, S5) could affirm the importance of the infectious CGMMV, localized in the 'early post-recovery stage' BYIs, to CGMMV systemic infection (Table 1). Importantly, the subsequent downregulation of JA signaling in the 'late post-recovery stage' BYIs was preceded by upregulation of the JA signaling inhibitors: SPL 9 and WRKY 51 already in the 'early post-recovery stage' BYIs (Supplementary Tables S6, S7). Apparently, under these circumstances, the reduced photosynthesis and activated JA signaling, which could both reduce SPL 9 via upregulation of miR156^36,37^, did not prevent SPL 9 upregulation thereby allowing its JA signaling inhibitory effect. Interestingly, upregulation of miR156 was detected at the pre-symptomatic stage of CGMMV inoculated cucumber plants grown at 27–32 °C^31^, supporting the role of JA signaling in the 'early post-recovery stage' BYIs. Our results apparently infer that the negative regulatory loops involving JA, miR156 and SPL 9^21,36,38^ are not yet fully understood and could also involve viral regulation of plant gene expressions^33,34^.

At the 'late post-recovery stage', pathway enrichment analyses of the BYIs, as compared to the associated dark green surrounding leaf tissues, showed downregulation of the important phenylpropanoid metabolic pathway associated with biosynthesis of the coumarins: scopoletin, scopolin and escolin (Table 2). Reduced expressions of six PAL genes, the key regulatory enzyme of the phenylpropanoid pathway, occurring in the 'late post-recovery stage' BYIs (Supplementary Table S8)^39^, indicate the important activity of the phenylpropanoid pathway in the associated dark green surrounding leaf tissues at that stage. Analyses of DEGs and pathway enrichment in the 'late post-recovery stage' dark green tissue compared to healthy cucumber plants showed upregulated expression of five PAL genes and an increase in biosynthesis of suberin monomers (involving the phenylpropanoid pathway) (Supplementary Table S9). Importantly, the set point of our study was not healthy plants but CGMMV-infected plants at a post-recovery stage, which would have a profound effect on the molecular pathways engaged by the plants in response to the reemerging disease. Nevertheless, the prominent upregulation of PAL in the 'late post-recovery stage' dark green tissue, when compared to healthy plants, further emphasized the tight association between activated phenylpropanoid pathway and plant antiviral response engaged in alleviating systemic infections. PAL suppression has already been associated with reduced systemic acquired resistance of *N. tabacum* plants against TMV^15^. Similarly, the importance of scopoletin in alleviating tobamovirus systemic infection via increasing ROI scavenging and decreasing viral replication (shown in protoplasts), was concluded in studies regarding salicylic acid-induced *N. tabacum* response to TMV^25^. In addition, a recent study on microRNA profile of CGMMV infected cucumber plants, grown at 27–32 °C, has shown at the symptomatic phase upregulation of a newly identified miRn1-3p, which targets a scopoletin glucosyltransferase-like gene^31,32^. Accordingly, downregulation of scopoletin glucosylation in TMV infected *N. tabacum* plants was associated with reduction of both scopoletin and its glucoside conjugated form scopolin, resulting in increased susceptibility to systemic TMV infection^25^. The above reports emphasize the crucial role of PAL downregulation in the increasing cucumber plant susceptibility to CGMMV systemic infection. Importantly, at the late post-recovery stage, the BYIs showed a concomitant increase in hydroxylated fatty acid biosynthesis pathway via the up-regulation of six 3-ketoacyl-CoA synthase genes (Supplementary Table S8) that could be associated with the upregulation of plant lipids. Upregulation of host plant lipids, shown to be engaged by positive-strand RNA viruses for replication^24^, could further support the identification of the 'late post-recovery stage' BYIs as the phenotypic marker for the molecular pathways associated with initiation of CGMMV systemic infection in cucumber plants. The phenylpropanoid pathway activation in 'late post-recovery stage' dark green tissue could comprise the initial molecular response active in DGIs that were identified in systemically infected plants as islands of low virus titer surrounded by virus infected areas^8^.

**Figure 5:**
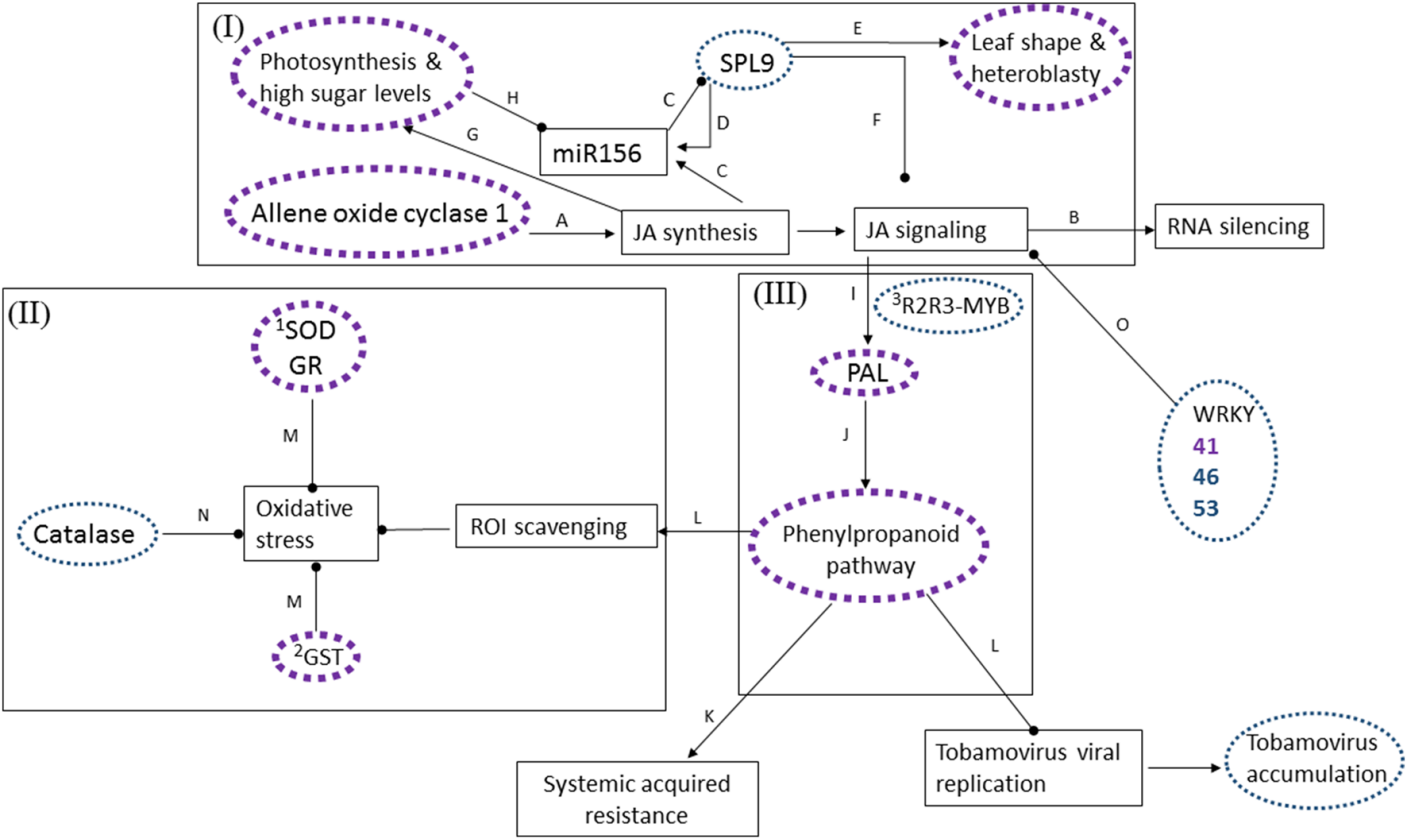
Molecular pathways differentially expressed in 'late post-recovery stage' bright yellow islands (BYIs) compared to the corresponding dark green surrounding tissues. (**I**) Molecular pathways associated with photosynthesis and vegetative leaf shape regulation are engaged in negative regulatory loops with jasmonic acid (JA) signaling, which could instigate RNA silencing and phenylpropanoid pathway activity. (**II**) Oxidative stress regulation by cellular antioxidant molecules: SOD, GR and GST, is reinforced by the phenolic coumarin products of the phenylpropanoid pathway. (**III**) JA signaling could upregulate the phenylpropanoid pathway, which is important for oxidative stress alleviation and induction of systemic acquired resistance response to tobamovirus infections. Molecular pathways and effectors differentially modified in 'late post-recovery stage' BYIs were circled. Molecular pathways presumed to be affected according to literature data were squared. Dotted purple colors mark downregulation effects; blue colors (small dots) mark upregulation effects. *SPL 9* SQUAMOSA promoter-binding-like protein 9, *JA* jasmonic acid, *PAL* phenylalanine ammonia lyase, *ROI* reactive oxygen intermediates, *SOD* superoxide dismutase, *GR* glutathione reductase, *GST* glutathione S-transferase. A^40^; B^14^; C^37^; D^38^; E^41^; F^21^; G^42^; H^36^; I^16^; J^39^; K^15^; L^25^; M^43^; N^44^; O^22^.

The principal molecular effectors and pathways, differentially expressed in the 'late post-recovery stage' BYIs concomitant with the downregulation of JA synthesis, apparently constitute a wide blockage exerted on JA signaling and oxidative stress alleviation (Fig. 5). Differential upregulation of several negative regulators of JA signaling, such as WRKY 51, SPL 9 and enzymes of the chloroplastic ascorbate–glutathione cycle: superoxide dismutase (SOD) and glutathione reductase (GR), preceded the initiation stage of systemic infection and were already detected in the 'early post-recovery stage' BYIs (Supplementary Tables S5-S7).

**Figure 6:**
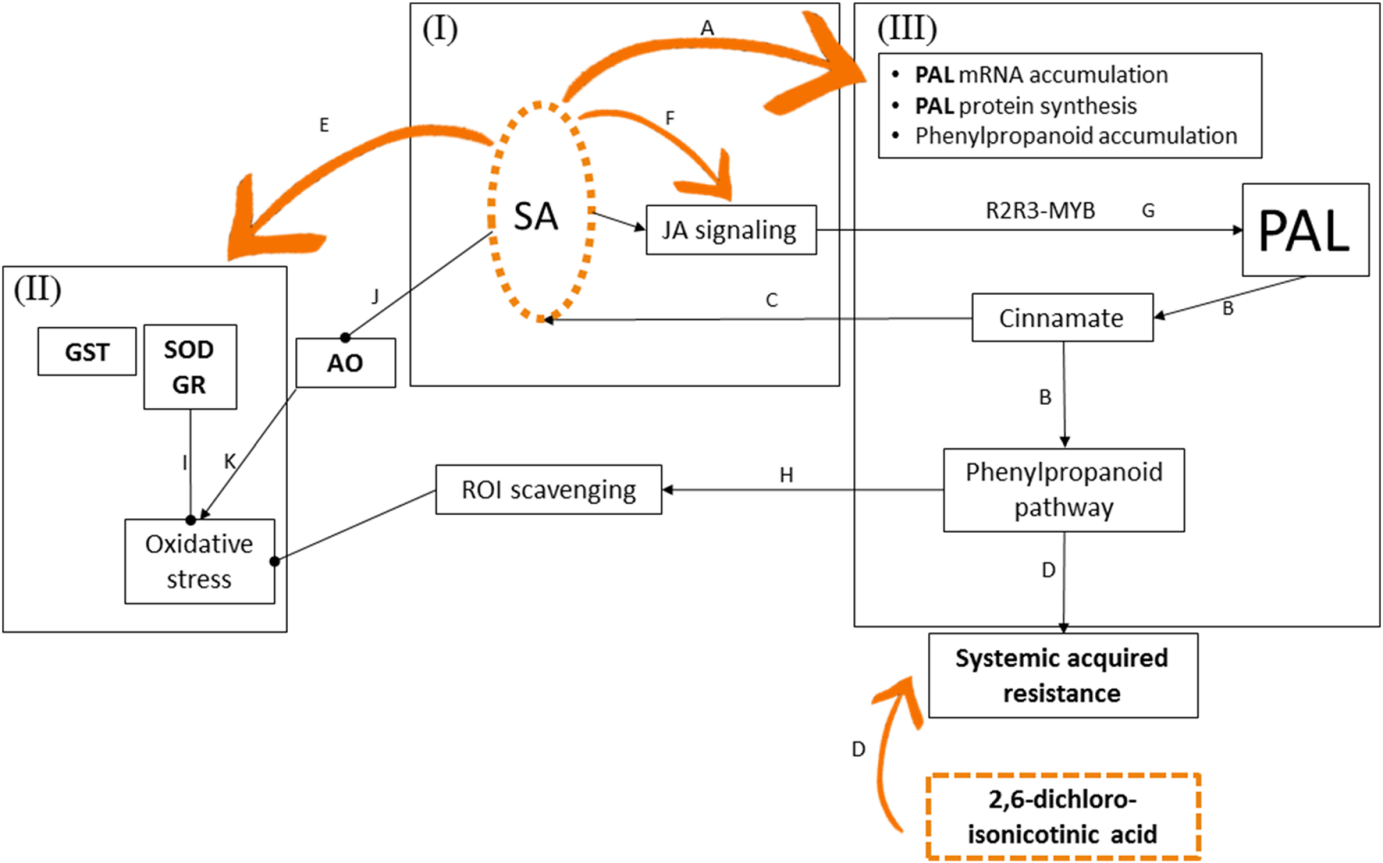
Molecular pathways and effectors susceptible to the proposed treatments with salicylic acid and 2,6-dichloro-isonicotinic acid. (**I**) SA upregulates PAL activity at three stages: PAL mRNA accumulation, PAL protein synthesis and phenolic compound accumulation. PAL involvement in synthesis of endogenous SA could accelerate the treatment effects. SA could also induce JA synthesis via a non-canonical pathway. (**II**) SA activation of SOD, GR and GST as well as its effect on downregulation of AO could alleviate oxidative stress associated with tobamovirus infections. (**III**) SA induced PAL could have a major effect on the phenylpropanoid pathway associated with ROI scavenging, thereby reducing oxidative stress, as well as instigation of systemic acquired resistance. Co-treatments with 2,6-dichloro-isonicotinic acid and SA, which additively activate systemic acquired resistance against tobamoviruses, could strengthen the SA effects. *SA* salicylic acid, *PAL* phenylalanine ammonia lyase, *JA* jasmonic acid, *SOD* superoxide dismutase, *GR* glutathione reductase, *GST* glutathione S-transferase, *AO* l-ascorbate oxidase, *ROI* reactive oxygen intermediates. A^45^; B^39^; C^46^; D^15^; E^19^; F^47^; G^16^; H^25^; I^43^; J^48^; K^14^.

Under these circumstances, a suggested early treatment of CGMMV-infected cucumber plants at the time-window of the BYI phenotype appearance, is primarily the salicylic acid (SA) application. SA could induce upregulation of the critical phenylpropanoid pathway, via PAL activation, as well as alleviation of oxidative stress via upregulation of SOD and GR and downregulation of AO, thereby circumventing the specific inhibitory activities exerted on JA signaling and its associated molecular effects (Fig. 6).

Combined activities of SA and JA have been shown to be essential for *N. benthamiana* systemic resistance against TMV^49^ and it has recently been shown that SA could promote JA signaling as well via a non-canonical pathway^47^. Importantly, an additive effect on amelioration of systemic acquired resistance against TMV in *N. tabacum* was observed by co-treatments with SA and 2,6-dichloroisonicotinic acid or its methyl ester^15^ (Fig. 6). Disclosing the cucumber molecular pathways, engaged at the early CGMMV disease initiation stages, have allowed pinpointing the preferable treatments in order to confine the CGMMV disease and prevent viral systemic infection. Applying various treatments on CGMMV-infected cucumber plants, showing the early post-recovery stage phenotypes, will promote understanding of the molecular mechanisms critical for the plant defense response.

## Materials and methods

### Cucumber plant cultivations and CGMMV inoculations

Cucumber seedlings from the commercial varieties Senyal (SN), Romi (RM), Kingstar (KS), Derby (DB), Samba (SA), Dingo (DN), Beit Alfa (BA), Noname, Ilan and the non-commercial variety 726 were planted in 0.5 L plastic pots and grown in a temperature-controlled glasshouse (3 m × 6 m) at 25 ± 2 °C. In order to prevent infections with insect-borne plant viruses and insect infestations, plants were irrigated with a mixture of two systemic insecticides, 0.01% CONFIDOR imidacloprid-based (BAYER) and 0.02% VERIMARK cyantraniliprole-based (DUPONT). The virus inoculum source was prepared from cucumber leaves of CGMMV-Rd inoculated plants (using frozen lyophilized material of CGMMV-Rd, GenBank accession No. KF155230) ground in sodium phosphate buffer (10 mM, pH = 7.0). Sequencing of CGMMV-Rd was done using viral RNA purified from CGMMV-Rd virion preparation that was fragmented by sonication and sequenced by SOLiD next generation sequencing (NGS) platform^50^, showing no other cucurbit infecting viruses in the preparation. CGMMV-Rd inoculum source was also tested negative for the presence of the vector transmitted viruses: cucumber mosaic virus (CMV), zucchini yellow mosaic virus (ZYMV), papaya ringspot virus (PRSV), cucurbit yellow stunting disorder virus (CYSDV), cucumber vein yellowing virus (CVYV) and cucurbit chlorotic yellows virus (CCYV) by RT-PCR prior to the preparation of an inoculum source. For CGMMV mechanical inoculations cucumber plants at the age of two true leaves were inoculated by dusting with carborundum the second true leaf that was hand-rubbed with CGMMV-Rd inoculum solution.

Studies of the effects of an abrupt temperature raise on CGMMV disease etiology were carried out by subjecting the various CGMMV inoculated cucumber cultivars and control un-inoculated plants, grown at 25 ± 2 °C, to an abrupt temperature raise at seven days post inoculation (dpi) before symptom development, reaching 32 ± 2 °C by fifteen min. In parallel, CGMMV inoculated plants were grown at constant temperatures of 25 °C and 32 °C. A close follow-up of symptom development was conducted on six of the cultivars. The experiments with SN plants were repeated four times (designated SN, SN2, SN3 and SN4) on 60, 11, 10 and 52 inoculated SN plants compared to 60, 5, 10 and 10 control un-inoculated SN plants, respectively. Cucumber cultivars: RM (10 inoculated vs 10 controls), KS (13 inoculated vs 3 controls), DB and SA (6 inoculated vs 5 controls of each *cv.*), as well as 726 (5 inoculated vs 5 controls) were included. Cucumber *cv.* Noname (10 inoculated vs 5 controls) was studied separately. The disease phenotype progression following the high temperature induced recovery, which was observed in response to an abrupt temperature raise, served for deduction of a time-table scheme postulating the manifestation of the new BYI phenotypes in field grown cucumber plants following an extreme diurnal temperature change. Symptom severity ratings that served the time-table scheme was determined phenotypically: 0–1: no visible symptoms; 2: bright yellow islands; 3–7: slight to medium severity showing a combined phenotype of yellow patched mottling; 7 and higher: severe symptoms of yellow patched mottling and mosaic on slightly deformed leaves associated with systemic CGMMV infection confirmed by ELISA test. CGMMV inoculated cucumber plants that were kept at the 25 ± 2 °C growth chamber were symptomatic but did not show the new BYI phenotypes (Fig. 1). Importantly, CGMMV inoculated plants that were grown at a constant 32 ± 2 °C growth chamber showed systemic infection that was followed by a recovery stage but the reemerging symptoms scarcely showed distinct BYI phenotypes (Supplementary Fig. S2). Meteorology data collected from the AgriMeteo website (http://www.meteo.co.il/home/), which provides access to recorded data from all meteorological stations in Israel, served for applying the CGMMV disease etiology scheme to predict the appropriate time-windows for manifestations of the unique disease BYI phenotypes in a commercial cucumber net-house. In addition, several additional commercial net-houses were screened for BYI phenotypes in unscheduled visits during early summer weeks.

Biological assays for the infectious potential of the BYIs and the associated dark green surrounding tissues of CGMMV inoculated cucumber plants, observed at a post-recovery stage following an abrupt temperature raise, were carried out by inoculating cucumber plants *cvs*. SN and Noname with inoculum sources prepared from the different phenotypes. The inoculum sources dissected from 'early post-recovery stage' leaves were unequally sampled. Five 3 mm discs, dissected from the 'early post-recovery stage' BYIs, and the whole dark green tissue surrounding the BYIs (~ 75 cm^2^, 1.4 gr) were used for inoculation of 10 *cv*. SN plants per inoculum source. The inoculum sources dissected from 'late post-recovery stage' BYIs and the associated dark green surrounding tissues were equally weighed (4 discs from each source, total weight of ~ 20 mg) and used for inoculation of 30 *cv*. Noname plants per inoculum source. In both early and late post-recovery stage bioassays the inoculated plants from each of the tested groups were divided between two different temperature controlled growth chambers of 25 °C and 32 °C.

### Temperature logging

Temperatures in the growing chambers were logged using a HOBO U12 Temp/RH Data Logger (Onset, USA). Four sensors were employed: two sensors were placed at each side of the chamber to record ambient air temperatures, while the other two were placed inside the growing medium of the plants at opposing sides of the control and the infected groups. Data were recorded at hourly intervals, extracted using HOBO-ware software and exported to Microsoft Excel format for further analyses.

### Viral RNA extractions and RT-PCR

Viral RNA was extracted from either the BYIs or the associated dark green surrounding tissues of early and late post recovery stages, using the AccuPrep Viral RNA Extraction Kit (Bioneer, KOR), according to the manufacturer's instructions. For the RT-PCR reactions, the viral RNA served as a template using either the RevertAid First Strand cDNA Synthesis Kit (Thermo Fisher Scientific, USA), with the R-3′*XhoI* primer designed for the CGMMV 3′ UTR(Reingold et al., 2013a) (5′-AAACTCGAGTGGGCCCCTACCCGGGGAA-3′; at position 6424 nt.)^6^, or the qPCRBIO cDNA Synthesis Kit, according to manufacturer's protocol (PCR Biosystems, London, UK). The obtained cDNA served as a template for PCR amplification using PCRBIO Taq DNA Polymerase Mix Red (PCR Biosystems) and specific primers for CGMMV: F-62: 5′-ATGGCAAACATTAATGAACAAAT-3′ and R-1115: 5′-AACCACACAGAAAACGTGGC-3′^6^.

### Western blot analyses

Cucumber leaf samples were subjected to total protein extractions using the urea-sodium dodecyl sulfate (SDS)-ß-mercaptoethanol (USB) extraction buffer (75 mM Tris–HCL pH 6.8, 9 M urea, 4.5% SDS, 7.5% ß-mercaptoethanol). Leaf discs (~ 3 mm in diameter) were dissected from both the BYIs and the associated dark green surrounding tissues and homogenized separately in the USB buffer, keeping a constant ratio of buffer volume per disc number (60 µl/3 combined discs for each preparation). Homogenization was carried out in 1.7 ml Eppendorf tubes using plastic sticks. Samples were heated to 97 °C for 15 min followed by centrifugation at 13,000 rpm for 15 min and the supernatant was added to Laemmli loading buffer^51^. The protein samples were separated on 15% SDS–polyacrylamide gel electrophoresis (PAGE) for 30 min at 40 V followed by 1.5 h at 100 V, and then electro-blotted for 30 min at 200 mAmp, onto a nitrocellulose membrane using a semidry transfer apparatus (Bio-Rad, California, USA). Membranes were blocked with 3% non-fat dry milk in PBS for 2 h and then incubated with a specific laboratory-produced primary antibodies (1:3500) for 40 min at room temperature and overnight at 4 °C. After four washes with PBS-T (0.05% v/v Tween-20 in PBS), the membranes were incubated with goat anti-rabbit alkaline phosphatase (AP) conjugated antibodies (1:5000 in PBS, Sigma-Aldrich, Missouri, USA), followed by four washes with PBS-T. An AP substrate (NBT/BCIP, Bio-Rad, California, USA) was used for detection of CGMMV-CP. In order to get high quality results of AP signal Ponceau S staining was carried out following the CP detection. The nitrocellulose membranes were stained with staining solution [0.1% (w/v) Ponceau S and 5% (v/v) acetic acid in PBS] for 5 min. The membranes were washed 3 times with PBS and scanned showing both the AP signal and the total loaded proteins.

### Indirect enzyme-linked immunosorbent assay (ELISA)

Cucumber leaves at various CGMMV disease progression stages were ground in 1 ml coating buffer (Bioreba, Reinach, CH) and tested using indirect ELISA^52^. Samples were analyzed in duplicates. Laboratory produced CGMMV antiserum, diluted to a ratio of 1:3000 in PBS-milk (2% non-fat milk powder in PBS), was added and the plates were incubated for 3 h at 37 °C. A commercial AP conjugated goat anti-rabbit (IgG) antibodies (Sigma-Aldrich, USA, diluted 1:5000 in PBS) were added and incubated for 3 h at 37 °C. P-nitro phenyl phosphate substrate (Sigma) was used at a concentration of 0.6 mg/ml for AP activity detection at 405 nm and 620 nm. Samples with optical density (OD) values of 2.5 times the NR or higher were considered as CGMMV positive.

### Total RNA extractions, high throughput sequencing (HTS) and bioinformatics analyses

Total RNA was extracted from CGMMV-infected cucumber plants *cv*. KS. Leaves from two plants exhibiting either the early or the late post-recovery stage symptoms (two plants per each stage) and two un-inoculated control plants were subjected to dissections of uniform discs, 3 mm in diameter. The discs were dissected from either the BYIs or the corresponding dark green surrounding tissues, in the symptomatic plants, and parallel dissections were performed on the control un-inoculated plants comprising a total of 10 samples for HTS. Total RNA was extracted from three combined discs, from each of the tested symptomatic features as well as the un-inoculated controls, using the GenElute Mammalian Total RNA Miniprep Kit (Sigma-Aldrich) according to the manufacturer's instructions. Discs were ground in the lysis buffer using plastic sticks in 1.7 ml Eppendorf vials. Total RNA was eluted into RNase free double-distilled water (DDW); DNA was removed using the DNA-free DNA Removal Kit (Thermo Fisher Scientific, Massachusetts, USA) according to the manufacturer's instructions and stored at − 80 °C. The extracted RNA served for library constructions using the TruSeq RNA Library Prep (Illumina, California, USA) commercial kit. RNA sequences containing poly-A tails were enriched using beads containing oligo-dT primers. The enriched RNA was used for constructions of cDNA libraries using random hexamers. HTS result cleanup was performed using Trimmomatic software^53^. The clean reads were searched for viruses using VirusDetect software version 1.7 and plant virus database, using default parameters of the software pipeline^54^. The assembly involved combining de novo assembly and mapping to plant virus references from GenBank with velvet^55^ and bwa for mapping reads^56^. For differential gene expression analyses clean reads were mapped to the cucumber genome Gy14 v2 using Bowtie 2 software^57^. Sample transcript quantifications were performed using RSEM software, and differential cucumber plant gene expression analyses were performed using DESeq 2^58,59^. The data presented in this study are openly available in NCBI Sequence Read Archive (SRA) under BioProject accession PRJNA701150. GO-enrichment analyses were performed by using Fisher's exact test with multiple testing corrections of FDR^60^. Pathway enrichment analyses^61^ were performed using Cucurbit Genomic Database (CuGenDB)^62^ http://cucurbitgenomics.org/.

### Quantitative RT-PCR (RT-qPCR)

CGMMV infected cucumber plants *cv*. NONAME, showing the newly identified early and late post-recovery stage symptoms, were tested for CGMMV and the RDR1 genes: *CsRDR1a*, *CsRDR1b* and *CsRDR1c* using RT-qPCR. Uniform 3 mm discs (3 discs) were dissected from either the BYIs or the associated dark green surrounding tissues of leaves showing early or late post-recovery stages (keeping an equal leaf sources for testing each of the phenotypes and the controls). Each symptomatic feature was dissected from 3 to 4 different plants. In addition, control uninfected healthy plants and CGMMV systemically infected cucumber plants were analyzed in parallel. For total RNA extractions, performed using GenElute Mamalian Total RNA Miniprep Kit (Sigma-Aldrich), the discs were homogenized in the lysis buffer using Geno/Grinder 2010 (SPEX SamplePrep) automated tissue homogenizer in 2 ml vials containing 2 magnetic beads. DNA was removed using the DNA-free DNA removal kit (Thermo Fisher Scientific, Massachussets, USA). RNA concentrations were measured by a spectrophotometer NanoDrop ND1000 (Thermo Scientific, Wilmington, USA). cDNA synthesis was performed on 140 ng total RNA using Verso cDNA Kit (Thermo Fisher Scientific, Epson, UK) with the oligo (dT) and specific primers (10 pmol/µl) and Random Hexamers. RT-qPCR was performed using the power Fast SYBR Green PCR master MIX (Applied Biosystems, Thermo Fisher Scientific, Vilnius, LT) and running was performed using the StepOnePlus (Applied Biosystems, Fisher Scientific Company, Ottawa, Ontario, CAN). The cucumber endogenous gene *TIP41* served as the reference gene^63^ and was analyzed with each of the tested genes. Primer sets used for the analyses were: (1) CGMMV-CP, F 5′-GTTTCGCTTCTCAGCTCCAC-3′ and R 5′-CGCGTCATCAGTACGCTTTA-3′. (2) *CsRDR1a* (GenBank accession no. KT316424), F 5′-CGTTCTCATGTTCTGCCGTA-3′ and R 5′-TTCGACCAACCGGTAGAAAC-3′. (3) *CsRDR1b* (GenBank accession no. KT316425), F 5′-TAACAGCCGTGGATGTACCA-3′ and R 5′-ATCGCTTCCAGAGCATTCAT-3′. (4) *CsRDR1c* (GenBank accession no. KT316426), F 5′-GCTACAAACCTGCACCAACA-3′ and R 5′-CTCCAAGACCATCGTTCACC-3′. (5) *TIP41* (GenBank accession no. GW881871), F 5′-CAACAGGTGATATTGGATTATGATTATAC-3′ and R 5′-GCCAGCTCATCCTCATATAAG-3′. The amplification reactions (performed in duplicates) contained cDNA reverse transcribed from 140 ng RNA in a 15 µl reaction mixture containing 4 µl 1:4 diluted cDNA, 3 pmols of each primer and 7.5 µl Absolute QPCR Sybr Green Mix (Thermo Fisher Scientific, Vilnius, LT). Reaction conditions were: 10 min at 95 °C (“hot start”) followed by 40 cycles of 3 s at 94 °C, 15 s at 60 °C, and 20 s at 72 °C. The quantitative analysis was performed using the StepOnePlus bio system (Applied Biosystems, Fisher Scientific Company, Ottawa, Ontario). Relative gene expression ratios were calculated using 2^−ΔΔCt^ method^64^. The percent amplification efficiency of each of the analyzed samples equaled: 1%. ΔCt was calculated by subtracting Ct of *TIP41* gene from the Ct of each of the tested genes in the samples. ΔΔCt was calculated by subtracting mean ΔCt of each of the tested genes in systemically infected symptomatic leaves from the ΔCt of the respective gene in each of the analyzed samples. Calculated 2^−ΔΔCt^ of the tested genes in each of the 3–4 analyzed leaves of a specific phenotype, indicating relative gene expression ratios in the tested samples, were combined and the mean of 3–4 samples [± standard deviation (SD) of the mean] per each of the tested phenotypes were graphed. The relative CGMMV expression ratios between BYIs and the associated dark green surrounding tissues was calculated by subtracting CGMMV ΔCt of dark green tissues from CGMMV ΔCt of the corresponding BYIs and the mean of three plants served for calculation of 2^−ΔΔCt^ ± SD.

### Ethical approval

we confirm that all methods complied with relevant institutional, national, and international guidelines and legislation in the methods section of the manuscript.

## Supplementary Information

Supplementary Information 1.

Supplementary Information 2.

Supplementary Information 3.

Supplementary Information 4.

Supplementary Information 5.

Supplementary Information 6.

Supplementary Information 7.

Supplementary Information 8.

## Data Availability

The data presented in this study are openly available in NCBI Sequence Read Archive (SRA) under BioProject accession PRJNA701150. https://www.ncbi.nlm.nih.gov/Traces/study/?acc=PRJNA701150&o=acc_s%3Aa.
