# Supplementary material for: New early phenotypic markers for cucumber green mottle mosaic virus disease in cucumbers exposed to fluctuating extreme temperatures
Source: Sci Rep. 2021 Sep 24;11:19060. doi: 10.1038/s41598-021-98595-4 (PMC8463606; doi:10.1038/s41598-021-98595-4)
Supplement: Supplementary file 5 — Supplementary Information 5. [file 41598_2021_98595_MOESM5_ESM.pdf]

Figure 2, original agarose gels

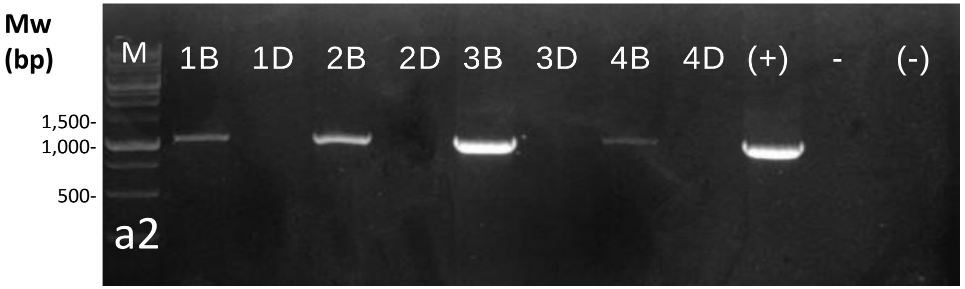

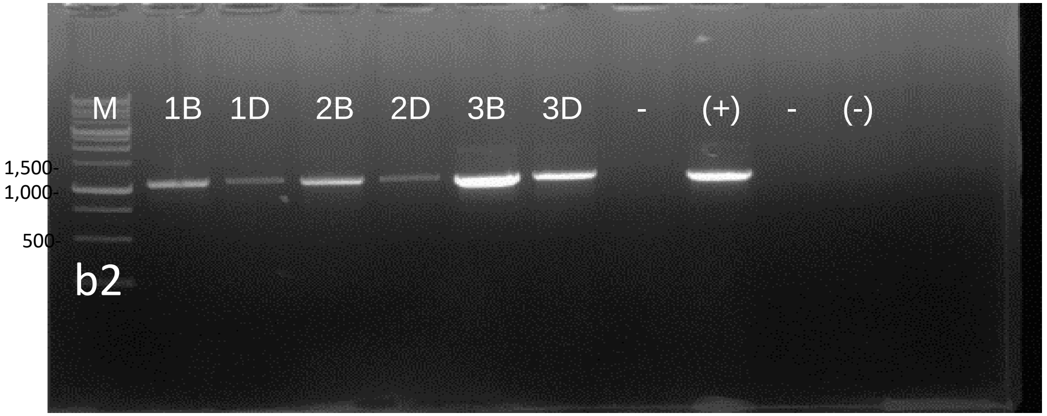

Figure 2, original western blot membranes

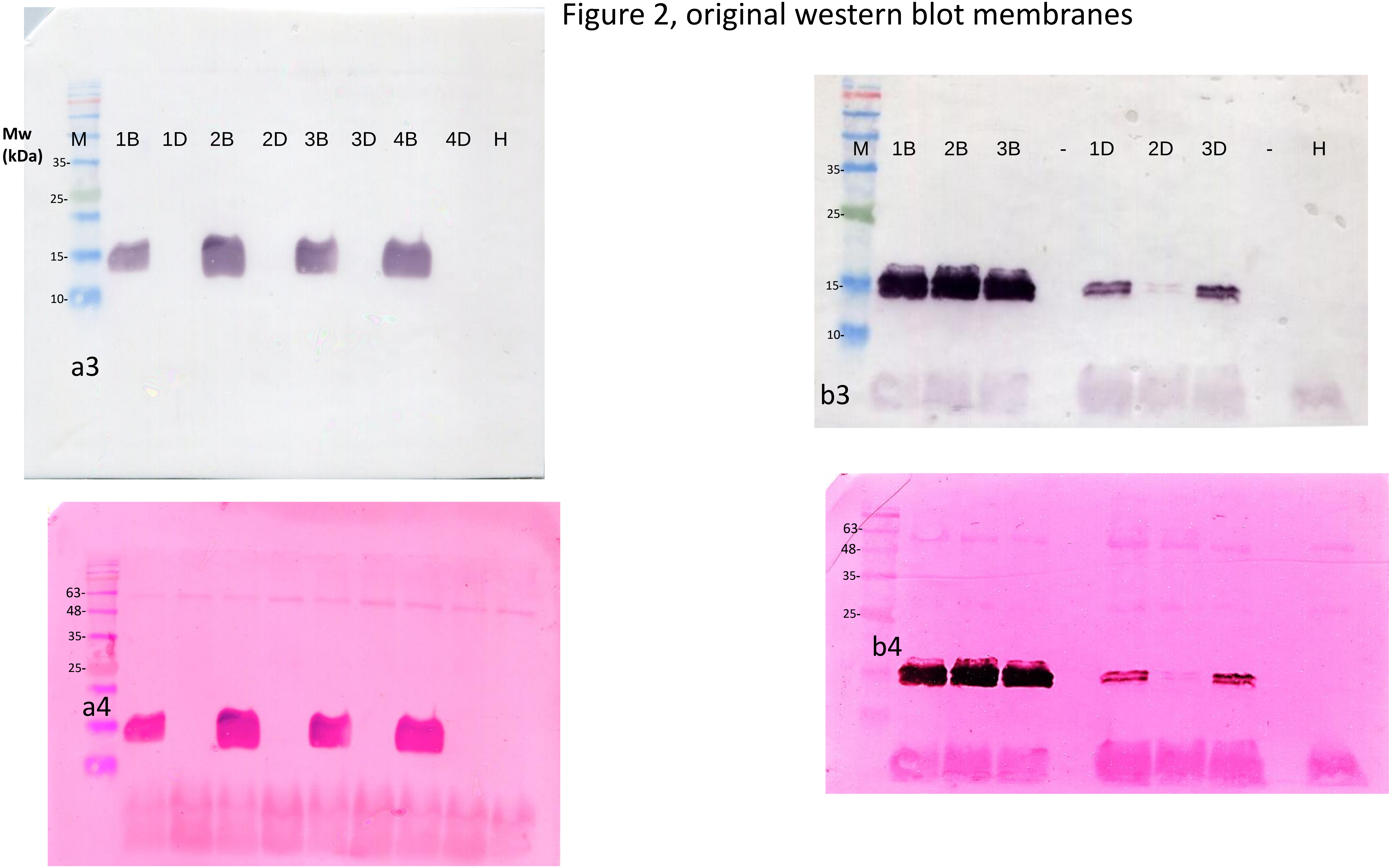

Figure 3, original membranes combined for the (e1)

(e1)

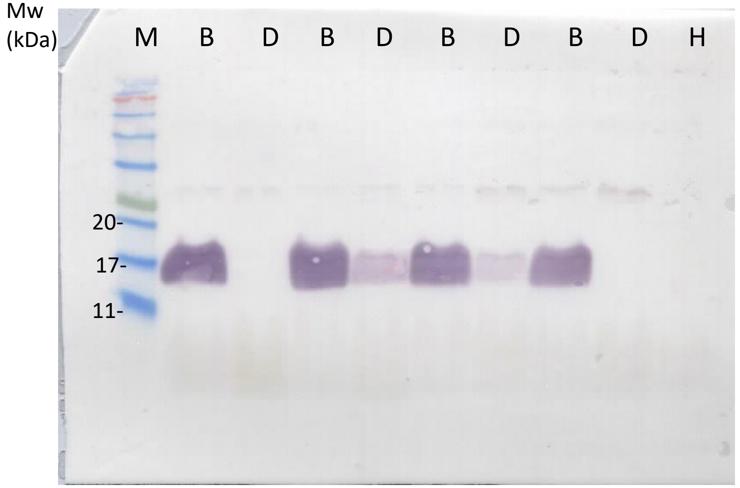

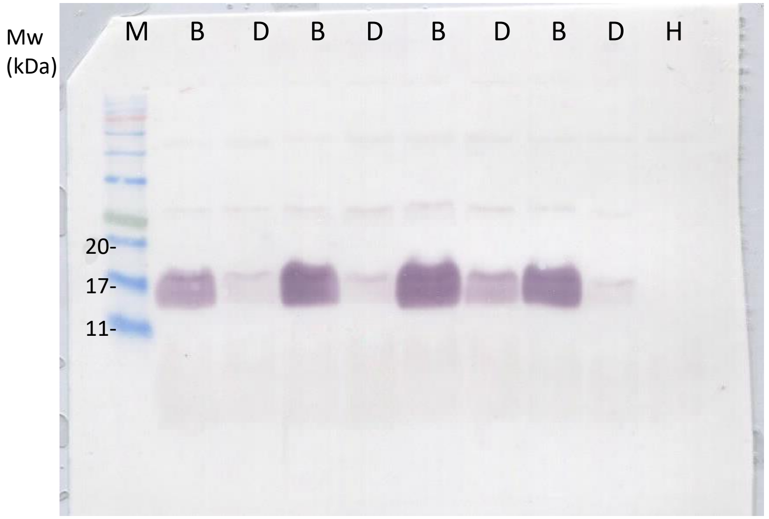

Figure 3, original membranes combined for the (e2)

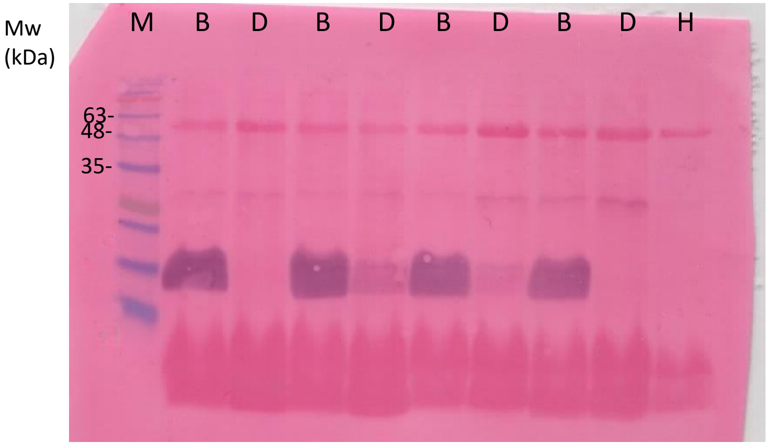

(e2)

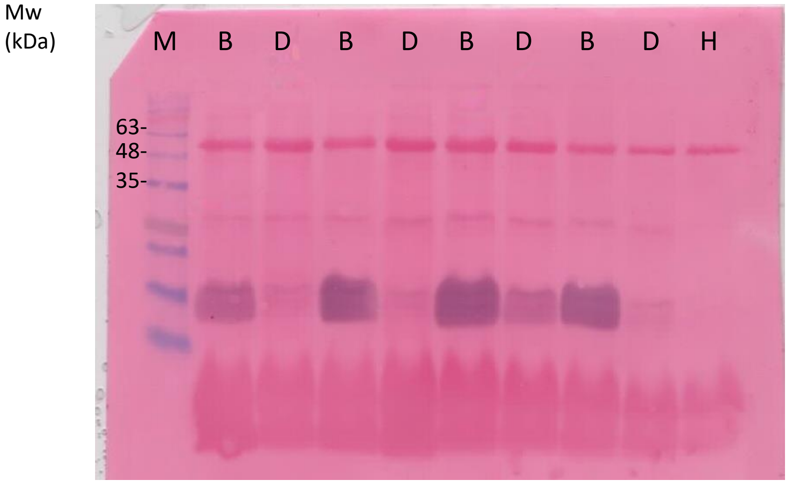

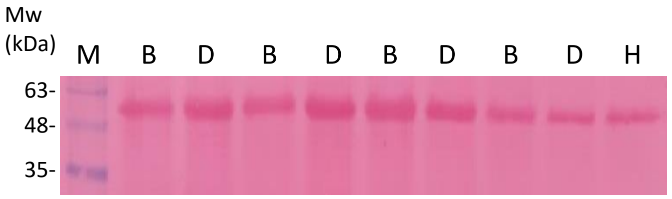
Mw
(kDa)
M B D B D B D B D H
63-
48-
35-

e1

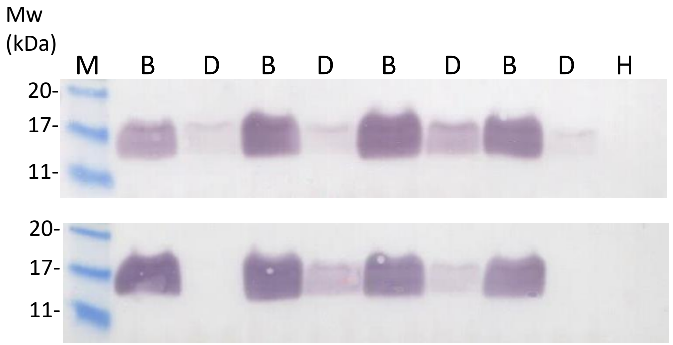
Mw
(kDa)
M B D B D B D B D H
20-
17-
11-
20-
17-
11-

e2

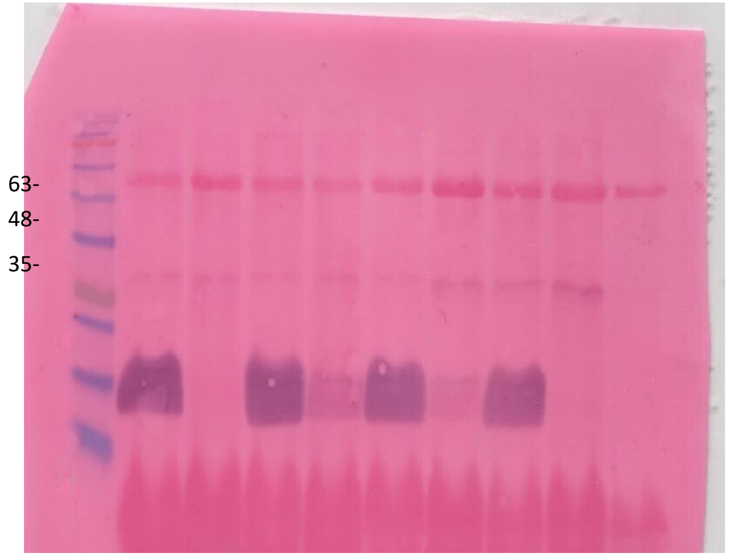
63-
48-
35-
