# Supplementary material for: New early phenotypic markers for cucumber green mottle mosaic virus disease in cucumbers exposed to fluctuating extreme temperatures
Source: Sci Rep. 2021 Sep 24;11:19060. doi: 10.1038/s41598-021-98595-4 (PMC8463606; doi:10.1038/s41598-021-98595-4)
Supplement: Supplementary file 6 — Supplementary Information 6. [file 41598_2021_98595_MOESM6_ESM.pdf]

New early phenotypic markers for cucumber green mottle mosaic virus disease in cucumbers exposed to fluctuating extreme temperatures

Ori Molad[1,2],
orimolad@gmail.com
Elisheva Smith[1],
elishevasmith@gmail.com

Neta Luria[1],
neta.luria.8@gmail.com

Noa Sela[1],
noa@volcani.agri.gov.il

Oded Lachman[1],
odedl@volcani.agri.gov.il

Elena Bakelman[1],
elenab@agri.gov.il

Diana Leibman[1]
diana@volcani.agri.gov.il

Aviv Dombrovsky[1*]
aviv@agri.gov.il

[1] Department of Plant Pathology and Weed Research, Agricultural Research Organization, The Volcani Center, 68 HaMaccabim Road, P.O.B 15159 Rishon LeZion 7505101, Israel.
[2] The Robert H. Smith Faculty of Agriculture, Food and Environment, The Hebrew University of Jerusalem, Rehovot 761001, Israel.
* Corresponding author
E-mail: aviv@volcani.agri.gov.il (AD)

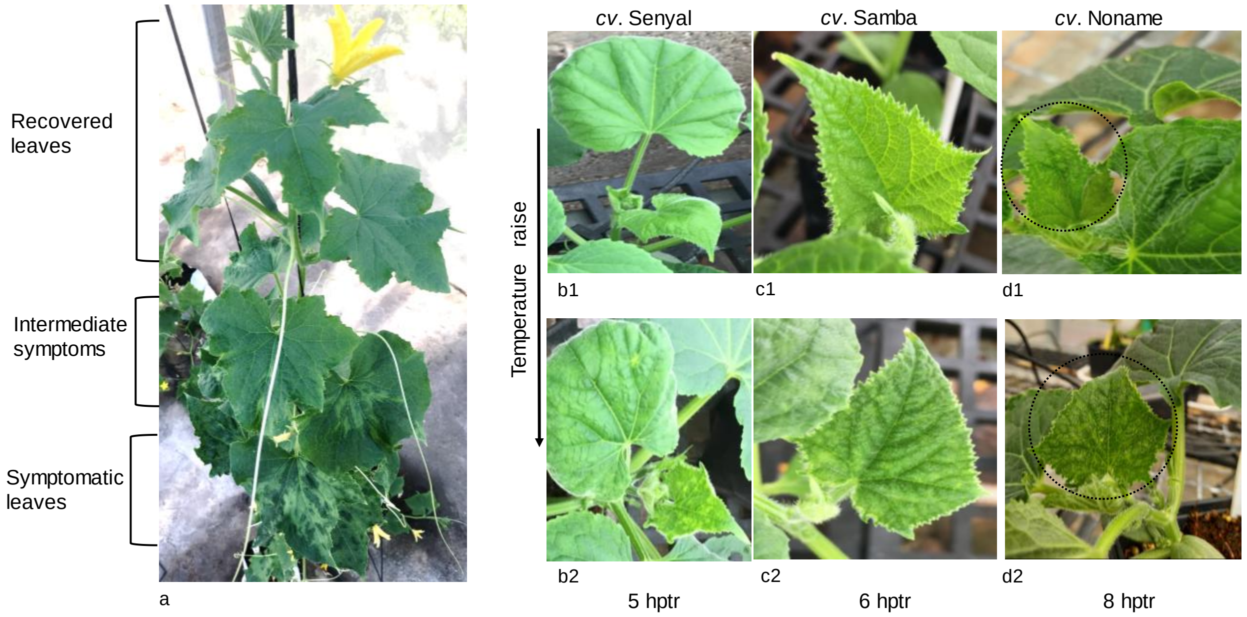

Supplementary Figure S1. Enhanced CGMMV disease symptom emergence and recovery in cucumbers upon an abrupt temperature raise from 25°C to 32°C. **(a)** A CGMMV infected cucumber plant *cv.* Ilan with symptomatic old leaves showing symptom recovery in upper leaves. **(b-d)** CGMMV inoculated cucumber plants grown at 25°C were exposed at a pre-symptomatic stage to an abrupt temperature raise to 32°C, reaching the high temperature in 15 min. Disease symptoms have appeared at different times following the temperature raise, depending on the cucumber cultivar, with Senyal, Samba and Noname cultivars showing the earliest occurrences. **(b1, c1, d1)** Phenotype of pre-symptomatic cucumber plants, grown at 25°C, before temperature raise. **(b2, c2, d2)** Symptom development of yellow patched mottling occurring in the cucumber upper leaves, below the apical leaf, 5-8 hours after temperature raise to 32°C. **(b1, 2)** *cv*. Senyal. **(c1, 2)** *cv*. Samba. **(d1, 2)** *cv*. Noname. A black dotted circle marks the same inspected leaf; hptr, hours post temperature raise.

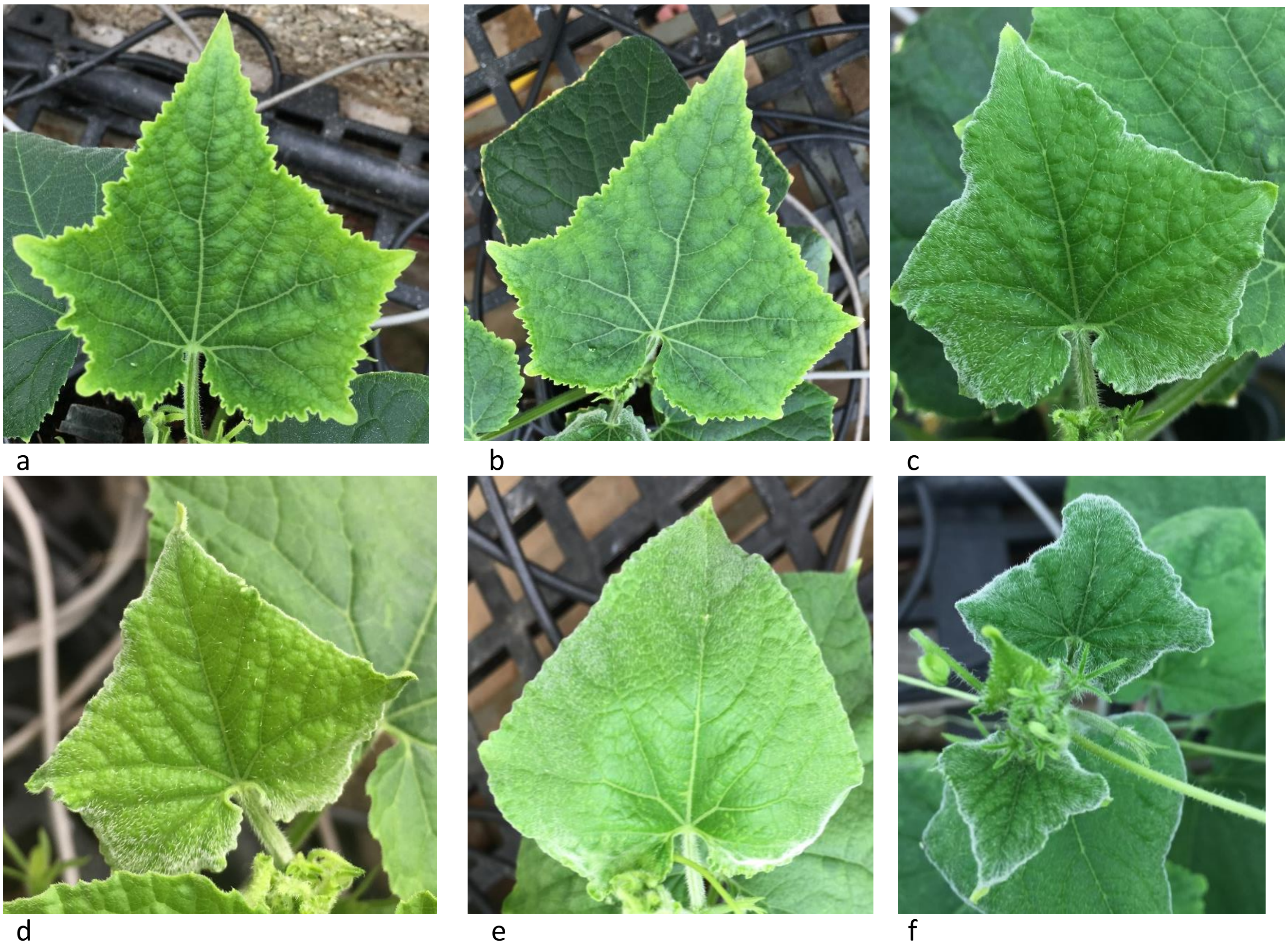

Supplementary Figure S2. CGMMV disease symptom development in cucumber plants grown at a constant high temperature. **(a-f)** A follow-up of CGMMV disease symptom manifestations in cucumber plants cv. Senyal, grown at a constant temperature of 32°C, preceding and following a recovery stage. **(a)** First appearance of mottling and mosaic symptoms at 10 dpi. **(b-f)** depict the CGMMV-infected plants at the sequential time points of 12, 15, 19, 23 and 27 dpi. dpi, days post inoculation. **(e)** Recovered plants, **(f)** Reemerging disease symptoms.

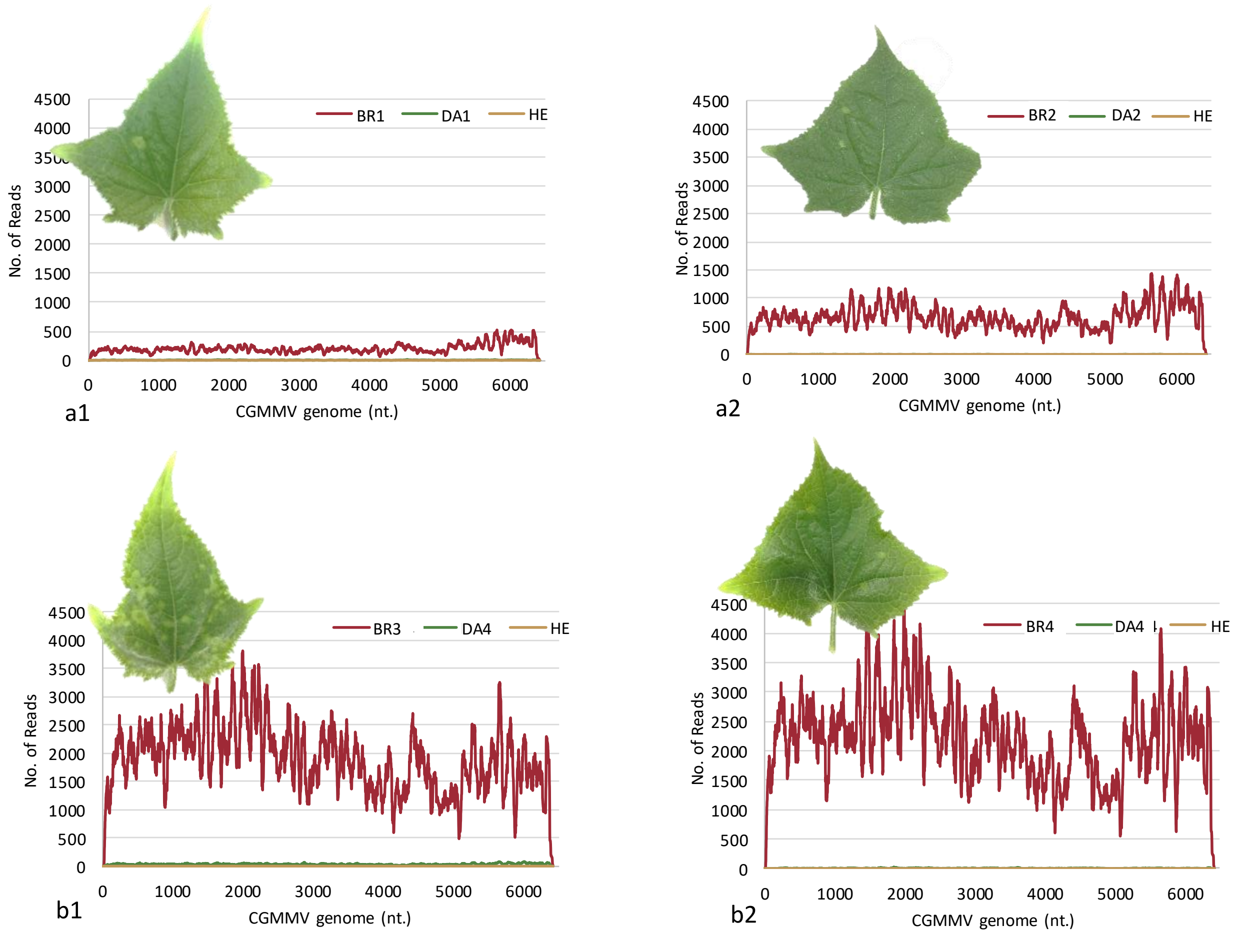

Supplementary Figure S3. High throughput sequencing of CGMMV in 'bright yellow islands' (BYIs) and the corresponding dark surrounding tissues at early and late post-recovery stages. **(a1, a2)** CGMMV genome coverage in cucumber leaf BYIs and the corresponding dark surrounding tissues of inoculated plants *cv*. Kingstar at an 'early post-recovery stage' compared to leaf samples of un-infected control plants. **(b1, b2)** CGMMV genome coverage in cucumber leaf BYIs and the corresponding dark surrounding tissues of inoculated plants *cv*. Kingstar at a 'late post-recovery stage' compared to leaf samples of un-infected control plants. BR, bright yellow islands; DA, corresponding dark surrounding tissues; HE, healthy un-infected controls.
