# Supplementary material for: New early phenotypic markers for cucumber green mottle mosaic virus disease in cucumbers exposed to fluctuating extreme temperatures
Source: Sci Rep. 2021 Sep 24;11:19060. doi: 10.1038/s41598-021-98595-4 (PMC8463606; doi:10.1038/s41598-021-98595-4)
Supplement: Supplementary file 7 — Supplementary Information 7. [file 41598_2021_98595_MOESM7_ESM.pdf]

New early phenotypic markers for cucumber green mottle mosaic virus disease in cucumbers exposed to fluctuating extreme temperatures

Ori Molad[1,2],
orimolad@gmail.com
Elisheva Smith[1],
elishevasmith@gmail.com

Neta Luria[1],
neta.luria.8@gmail.com

Noa Sela[1],
noa@volcani.agri.gov.il

Oded Lachman[1],
odedl@volcani.agri.gov.il

Elena Bakelman[1],
elenab@agri.gov.il

Diana Leibman[1]
diana@volcani.agri.gov.il

Aviv Dombrovsky[1*]
aviv@agri.gov.il

[1] Department of Plant Pathology and Weed Research, Agricultural Research Organization, The Volcani Center, 68 HaMaccabim Road, P.O.B 15159 Rishon LeZion 7505101, Israel.
[2] The Robert H. Smith Faculty of Agriculture, Food and Environment, The Hebrew University of Jerusalem, Rehovot 761001, Israel.
* Corresponding author
E-mail: aviv@volcani.agri.gov.il (AD)

Supplementary Table S2. Library quality prepared from early and late post-recovery stage BYIs and the corresponding dark surrounding tissues and CGMMV genome sequencing.

			***Virus assembly**		
Library source	Total reads	Read No. after quality cleaning and trimming	Contig numbers	Coverage (%)	Depth
'Early' BYIs (1)	14,432,619	14,300,352 (99.08%)	2	6,425 (99.8)	14.3
'Early' BYIs (2)	13,790,249	13,670,104 (99.13%)	3	6,426 (99.8)	136.0
'Late' BYIs (3)	14,205,302	14,036,668 (98.81%)	2	6,424 (99.8)	156.0
'Late' BYIs (4)	17,182,984	17010432 (99.00%)	3	6,426 (99.8)	38.4
'Early' Dark (1)	16,012,012	15,881,337 (99.18%)	1	228 (86.4)	0.3
'Early' Dark (2)	14,213,038	14,039,355 (98.78%)	5	6,413 (99.6)	2.4
'Late' Dark (3)	13,870,614	13,723,660 (98.94%)	12	1,458 (88.5)	0.4
'Late' Dark (4)	15,512,205	15,376,391 (99.12%)	-	-	-
Un-inoculated (He1)	16,742,039	16,577,843 (99.02%)	-	-	-
Un-inoculated (He2)	17,860,987	17,663,001 (98.89%)	-	-	-

'Early', 'early post-recovery stage'; 'Late', 'late post-recovery stage'; BYIs, bright yellow islands; Dark, corresponding dark surrounding tissues; He, healthy; in brackets, respective plant source for 'Early' and 'Late' stages and un-inoculated healthy controls; Contig, a contiguous DNA segment of overlapping reads; Coverage, % of the genome sequenced; Depth, an average no. of times a nucleotide in reference genome is covered by the sample reads; * data refer to de novo assembly and mapping to plant virus references, showing CGMMV genome sequencing.

Supplementary Table S5. Differential expression of genes involved in alleviating oxidative stress in bright yellow islands (BYIs) of early and late post-recovery stages compared to the corresponding dark surrounding tissues.

'Early post-recovery stage' BYIs' downregulated DEGs	'Early post-recovery stage' BYIs' upregulated DEGs	'Late post-recovery stage' BYIs' downregulated DEGs	'Late post-recovery stage' BYIs' upregulated DEGs
Thioredoxin (6)	-	Thioredoxin (5)	-
L-ascorbate oxidase (1)	L-ascorbate oxidase (2)	L-ascorbate oxidase (1)	L-ascorbate oxidase (4)
Superoxide dismutase (1)	-	Superoxide dismutase (2)	Superoxide dismutase (1)
Glutathione reductase (1)	-	Glutathione reductase (1)	-
-	Catalase and Catalase activity (2)	-	Catalase and Catalase activity (2)
Glutathione S-transferase (3)	Glutathione S-transferase (1)	Glutathione S-transferase (5)	Glutathione S-transferase (1)
Glutathione peroxidase (2)	Glutathione peroxidase (1)	Glutathione peroxidase (1)	-

DEGs, differentially expressed genes; in brackets, number of DEGs.

Supplementary Table S6. SQUAMOSA promoter-binding-like (SPL) proteins upregulated in the bright yellow islands (BYIs) of early and late post-recovery stages compared to the corresponding dark surrounding tissue.

Post-recovery stage BYIs	Gene description	*miR156-negatively regulated
Early	SQUAMOSA promoter binding protein (1)	-
	SPL 6 (1)	+
	SPL 8 (1)	-
	SPL 9 (1)	+
	SPL 13A (2)	+
	SPL 14 (1)	-
Late	SQUAMOSA promoter binding protein (1)	-
	SPL 6 (1)	+
	SPL 8 (1)	-
	SPL 9 (1)	+
	SPL 13A (2)	+

In brackets, no. of differentially expressed genes; * micro RNA156.

Supplementary Table S7. WRKY transcription factors differentially expressed in bright yellow islands (BYIs) of early and late post-recovery stages compared to the corresponding dark surrounding tissues.

Early post-recovery stage BYIs' downregulated genes	Early post-recovery stage BYIs' upregulated genes	Late post-recovery stage BYIs' downregulated genes	Late post-recovery stage BYIs' upregulated genes
-	WRKY-2 (1)	WRKY-26 (1)	[a]WRKY-12 (1)
-	[a]WRKY-12 (1)	WRKY-40 (1)	WRKY-13 (1)
-	[a]WRKY-15 (2)	[b]WRKY-41 (1)	[a]WRKY-15 (1)
-	WRKY-22 (1)	WRKY-44 isoform x2 (1)	WRKY-21 (1)
-	WRKY-31 (1)		WRKY-40 (1)
-	WRKY-32 (1)		[b]WRKY-46 (1)
-	WRKY-48 (1)		[b]WRKY-53 (1)
-	[b]WRKY-51 (1)		
-	WRKY-75 (1)		

In brackets, no. of differentially expressed genes; aWRKY genes differentially upregulated in BYIs of both early and late post-recovery stages; bWRKY transcription factors inhibiting jasmonic acid signaling.
